# Local senolysis in aged mice only partially replicates the benefits of systemic senolysis

**DOI:** 10.1172/JCI162519

**Published:** 2023-04-17

**Authors:** Joshua N. Farr, Dominik Saul, Madison L. Doolittle, Japneet Kaur, Jennifer L. Rowsey, Stephanie J. Vos, Mitchell N. Froemming, Anthony B. Lagnado, Yi Zhu, Megan Weivoda, Yuji Ikeno, Robert J. Pignolo, Laura J. Niedernhofer, Paul D. Robbins, Diana Jurk, João F. Passos, Nathan K. LeBrasseur, Tamara Tchkonia, James L. Kirkland, David G. Monroe, Sundeep Khosla

**Affiliations:** 1 Robert and Arlene Kogod Center on Aging,; 2 Division of Endocrinology,; 3 Department of Physiology and Biomedical Engineering, and; 4 Department of Hematology, Mayo Clinic College of Medicine, Rochester, Minnesota, USA.; 5 Department of Pathology and Laboratory Medicine, University of Texas Health Science Center, San Antonio, Texas, USA.; 6 Department of Medicine, Mayo Clinic College of Medicine, Rochester, Minnesota, USA.; 7 Institute on the Biology of Aging and Metabolism, Department of Biochemistry, Molecular Biology and Biophysics, University of Minnesota, Minneapolis, Minnesota, USA.; 8 Department of Physical Medicine and Rehabilitation, Mayo Clinic College of Medicine, Rochester, Minnesota, USA.

**Keywords:** Aging, Bone Biology, Bone disease, Cellular senescence, Osteoporosis

## Abstract

Clearance of senescent cells (SnCs) can prevent several age-related pathologies, including bone loss. However, the local versus systemic roles of SnCs in mediating tissue dysfunction remain unclear. Thus, we developed a mouse model (*p16-LOX-ATTAC*) that allowed for inducible SnC elimination (senolysis) in a cell-specific manner and compared the effects of local versus systemic senolysis during aging using bone as a prototype tissue. Specific removal of Sn osteocytes prevented age-related bone loss at the spine, but not the femur, by improving bone formation without affecting osteoclasts or marrow adipocytes. By contrast, systemic senolysis prevented bone loss at the spine and femur and not only improved bone formation, but also reduced osteoclast and marrow adipocyte numbers. Transplantation of SnCs into the peritoneal cavity of young mice caused bone loss and also induced senescence in distant host osteocytes. Collectively, our findings provide proof-of-concept evidence that local senolysis has health benefits in the context of aging, but, importantly, that local senolysis only partially replicates the benefits of systemic senolysis. Furthermore, we establish that SnCs, through their senescence-associated secretory phenotype (SASP), lead to senescence in distant cells. Therefore, our study indicates that optimizing senolytic drugs may require systemic instead of local SnC targeting to extend healthy aging.

## Graphical abstract

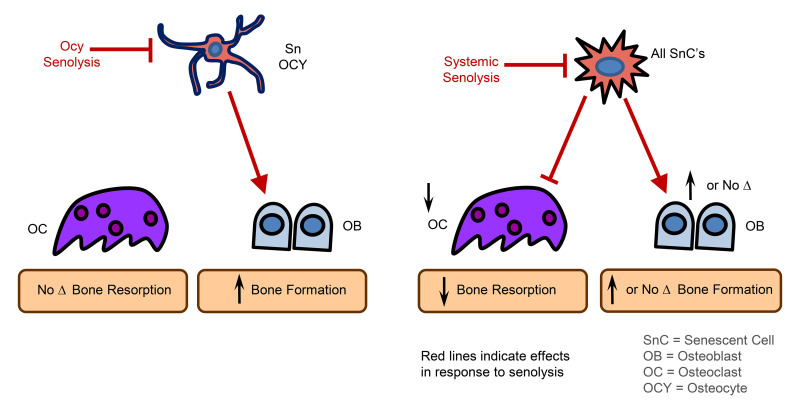

## Introduction

The aging population and life expectancy continue to increase (1), and while unlikely to reverse course, this trend of more people living longer creates challenges that society remains poorly equipped to handle, including a myriad of late-life chronic diseases and geriatric syndromes, which cluster in older individuals (2). However, rather than extending life span at all costs, compressing the period of late-life morbidity would not only have tremendous economic value (3), but also allow elderly people to maintain autonomy, independence, and well-being in old age (4). Because hallmarks of aging promote age-related diseases (5, 6), therapeutic interference with fundamental aging mechanisms represents an attractive strategy to compress late-life morbidity (7).

In this context, cellular senescence, a permanent state of cell-cycle arrest induced by various stressors to halt the proliferation of damaged or dysfunctional cells, has emerged as a key target for therapeutic exploitation to prevent age-related pathologies (8). Senescent cells (SnCs) develop an inflammatory and tissue-destructive secretome to prompt their immune-mediated clearance, but which can also drive chronic sterile inflammation and loss of tissue homeostasis and cause aging and age-related diseases (8). Transgenic mouse models have been used to demonstrate that elimination of SnCs (i.e., senolysis) is therapeutic, able to extend healthspan during chronological aging, and reduces the severity of multiple age-related pathologies (9, 10). These findings inspired the discovery of a class of drugs known as senolytics that selectively kill SnCs as a therapeutic approach to extend healthy aging (11). However, as emerging data continue to reinforce the premise that a single senolytic may not be capable of systemically clearing all SnC types (12), it has become of paramount importance to understand the local versus systemic effects of eliminating SnCs. Indeed, elucidating the cell-autonomous versus -nonautonomous roles of specific SnCs in mediating tissue dysfunction will help guide the development of senolytics (or their combinations) that most broadly alleviate age-related diseases as a group.

In the present study, we hypothesized that bone would arguably represent the ideal tissue in which to address this issue, with a focus on the osteocyte, for multiple reasons: (a) unlike the heterogeneity of most tissues, bone is abundantly populated by osteocytes (comprising >95% of all skeletal cells) (13); (b) spatial and temporal control of precise gene manipulations in osteocytes using the *Cre-loxP* system is facilitated by a well-characterized *Cre* line (i.e., *8kb DMP1-Cre*) (14); and (c) along with a host of other crucial functions (15), the osteocyte operates in a network to precisely control the actions of other skeletal cells, including osteoclasts and osteoblasts, to execute bone remodeling.

Indeed, signals arising from osteocytes, acting via the RankL/OPG and Sost/Dkk1/Wnt axes, normally maintain the balance between bone resorption and formation (15). However, these tightly coordinated events go awry in old age through mechanisms that are not fully understood, leading to a net imbalance between bone resorption and bone formation (16). Multipotent mesenchymal stem cells (MSCs) are the source of osteoblasts; however, because of cell-autonomous aging mechanisms or in response to nonautonomous aging signals derived from unknown sources, e.g., dysfunctional osteocytes (17), these progenitors preferentially differentiate into bone marrow adipocytes in old age (16). Therefore, defective bone formation and increased marrow adiposity are hallmarks of age-related osteoporosis.

Aging bone contains cells with markers of senescence, which, as noted earlier, is a stress response program activated by cyclin-dependent kinase inhibitors, most notably *p16^Ink4a^* (encoded by *Cdkn2a*) and *p21^Cip1^* (encoded by *Cdkn1a*) (18, 19). With aging, SnCs have increased expression of *p16^Ink4a^* in several mammalian tissues (20), whereas *p21^Cip1^* seems to be induced by cells under acute duress leading to senescence in other circumstances (21), including tissue repair (22, 23). Additional molecular properties of SnCs include DNA double-stranded breaks at sites of telomeres (i.e., telomere-associated foci [TAF]) (24) and, in response to this permanent damage, an upregulated transcriptional machinery to generate a robust secretome composed of numerous biologically active molecules known as the senescence-associated secretory phenotype (SASP). These and other senescence markers have been used by multiple groups to identify SnCs in the bone microenvironment with aging (25, 26). To establish whether systemic senolysis prevents age-related bone loss, our group eliminated *p16^Ink4a+^* SnCs using *p16-INK-ATTAC* (apoptosis through targeted activation of caspase 8) — a “suicide” transgene driven by the *p16^Ink4a^* promoter (9). Activation of the *p16^Ink4a^*-driven suicide transgene over a 4-month period in old mice prevented age-related trabecular and cortical bone loss at the spine and femur by reducing bone resorption, improving bone formation, and suppressing bone marrow adiposity (27). Thus, systemic clearance of *p16^Ink4a+^* SnCs has both antiresorptive and anabolic effects on aging bone and reduces bone marrow adipocytes (28). However, the precise roles of bone-resident versus nonskeletal SnCs in mediating age-related bone loss remain unclear.

To permit the investigation of cell-specific SnC elimination, we developed and used a mouse model (*p16-LOX-ATTAC*) harboring a “suicide” transgene that allows for inducible senolysis in a cell-specific manner. By comparing local (cell-specific) senolysis with systemic senolysis in transgenic mouse models, and through complementary SnC transplantation studies, we dissected the cell-autonomous versus -nonautonomous effects of SnCs using the skeleton as a prototype tissue.

## Results

### Sn osteocytes accumulate in bone with advancing age.

**Figure 1:**
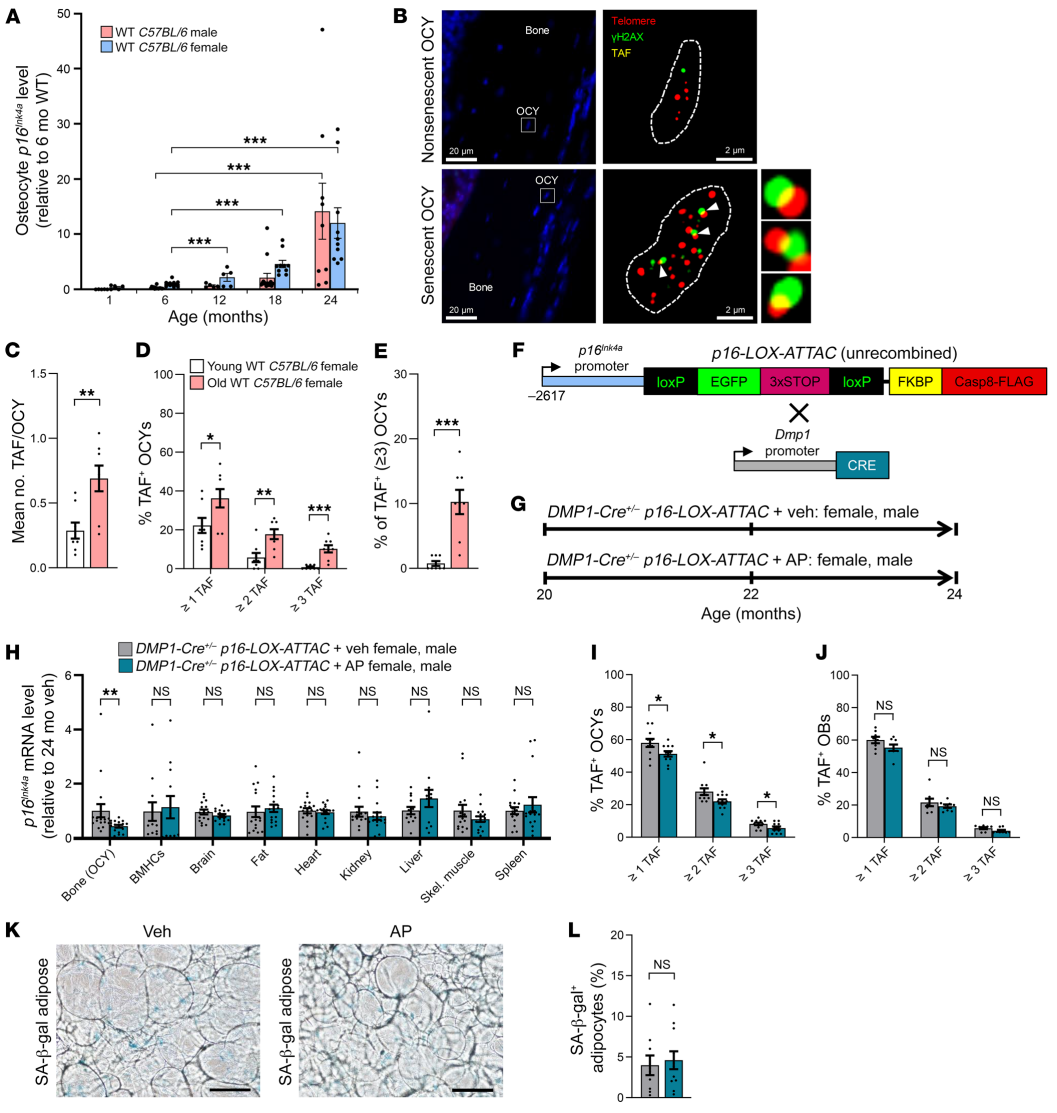
Sn osteocytes accumulate in bone with aging and are cleared by AP treatment in old *DMP1-Cre^+/–^* *p16-LOX-ATTAC* mice. (**A**) Changes in female (pink bars) and male (blue bars) murine *p16^Ink4a^* mRNA expression throughout life in osteocyte-enriched bone from *C57BL/6* WT mice, relative to young adult (6-month-old) mice. ***P < 0.001, by ANOVA with Tukey’s post hoc test. (**B**) Representative images of a non-Sn osteocyte (original magnification, ×63 oil) in a young (6-month-old) mouse versus a Sn osteocyte in an old (24-month-old) mouse according to the TAF (white arrows) assay (*n* = 8 females/group). Scale bars: 20 μm and 2 μm (enlarged insets). (**C**–**E**) Quantification of (**C**) the mean TAF/osteocyte and (**D**) the mean percentage of TAF^+^ osteocytes/mouse based on: the percentage of osteocytes with 1 or more TAF, the percentage of osteocytes with 2 or more TAF, and the percentage of osteocytes with 3 or more TAF, respectively; and quantification of (**E**) the mean percentage of 3 or more TAF^+^ osteocytes/mouse (*n* = 8 females/group). (**F**) Schematic of the unrecombined *p16-LOX-ATTAC* construct and cross with *DMP1-Cre* mice. (**G**) Study design for local clearance of Sn osteocytes in old (20 months) *p16-LOX-ATTAC* x *DMP1-Cre* mouse cohorts, males and females combined, randomized to Veh (gray) or AP (teal) treatment for 4 months. (**H**) RT-qPCR analysis of *p16^Ink4a^* mRNA expression across tissues in mice (males and females combined, *n* = 12–18 per tissue) treated with Veh (gray) or AP (teal). (**I** and **J**) Quantification of (**I**) the mean percentage of TAF^+^ osteocytes/mouse and (**J**) the mean percentage of TAF^+^ osteoblasts per mouse (OBs/mouse) based on the percentage of cells with 1 or more TAF, the percentage of cells with 2 or more TAF, and the percentage of cells with 3 or more TAF, respectively. (**K**) Representative images of perigonadal adipose tissue staining for SA–β-Gal^+^ cells in Veh- and AP-treated mice. Scale bars: 100 μm. (**L**) Quantification of SA–β-Gal^+^ adipocytes in mice treated with Veh (gray, *n* = 9: *n* = 5 females, *n* = 4 males) or AP (teal, *n* = 11: *n* = 6 females, *n* = 5 males). Data represent the mean ± SEM. *P < 0.05, **P < 0.01, and ***P < 0.001, by independent samples Student’s *t* test or Wilcoxon rank-sum test, as appropriate. OCY, osteocytes.

As an extension of our previous studies (26, 27), we first verified that Sn osteocytes accumulate throughout the life span of C57BL/6 WT mice up to age 24 months, a time when age-related bone loss in both female and male mice is well established. Mice were sacrificed at 1, 6, 12, 18, and 24 months of age and highly enriched cell preparations of osteocytes were isolated (see Methods) at each time point. real-time quantitative PCR (RT-qPCR) analysis confirmed that *p16^Ink4a^* transcript levels increased in murine osteocyte–enriched bone samples with advancing age in both sexes when analyzing females and males separately, albeit at somewhat different rates (Figure 1A) as well as in both sexes combined (Supplemental Figure 1A; supplemental material available online with this article; https://doi.org/10.1172/JCI162519DS1). In addition, as previously shown by our group (26), osteocyte-enriched bones from old mice displayed significant upregulation of SASP components, including chemokines, inflammatory cytokines, and matrix metalloproteases (Supplemental Figure 1B). To establish senescence at the single-cell level, we performed TAF staining (Figure 1B), a measure of DNA damage (γ-H2AX) colocalized with telomeres, of osteocytes because TAFs are perhaps the most definitive marker of cellular senescence (24). This assay revealed a highly significant accumulation of Sn osteocytes with aging in old (24 months old) compared with young adult (6 months old) mice (Figure 1, C–E). Given that osteocytes are crucial sources of signaling factors in the coordination of bone remodeling (15), and based on our findings here that at least a subset of osteocytes undergoes senescence and develops a robust SASP with aging, we hypothesized that Sn osteocytes are key drivers of age-related bone loss. To test this hypothesis, we developed and used a mouse model, *p16-LOX-ATTAC*, which allows for the inducible elimination of specific SnC types when crossed with a cell-specific *Cre*-recombinase (*Cre*) model, e.g., *8kb* *DMP1-Cre* for osteocytes (14).

### Development and validation of a model for eliminating specific SnC types.

In order to permit tissue-specific expression of *ATTAC* (FKBP-Casp8) (29), the same suicide transgene driven by the *p16^Ink4a^* promoter in *p16-INK-ATTAC* mice (9, 10), activated upon administration of a synthetic drug, AP20187 (hereafter referred to as AP), was placed downstream of a *p16^Ink4a^* promoter-*EGFP* cassette with 3 SV40 poly(A) transcription termination sequences (*3xSTOP*) flanked by *loxP* sites (Figure 1F). In the unrecombined state, mice harboring this transgene express *EGFP*, but not the FLAG-tagged FKBP-Casp8 fusion protein. However, when crossed with a cell-specific *Cre*, the *EGFP* and *3xSTOP* cassette are removed, resulting in expression of the FLAG-tagged FKBP-Casp8 fusion protein. Thus, in cells with active *Cre*, there is no *EGFP* expression, but the FLAG-tagged FKBP-Casp8 transgene is expressed. Administration of AP to *p16-LOX-ATTAC* mice carrying a cell-type–specific *Cre* driver leads to activation of the FKBP-Casp8 apoptosis cascade in a specific SnC type to allow for their inducible “suicide.” Importantly, the combination of cell-specific *Cre* expression and AP permits both cell-specific and temporal (e.g., in aged mice) control of SnC elimination.

To first validate this model, we isolated bone marrow stromal cells (BMSCs) from *p16-LOX-ATTAC* mice (unrecombined; Supplemental Figure 2A) or *p16-LOX-ATTAC* mice crossed with the ubiquitous *CMV-Cre* (Supplemental Figure 2B), which deletes the *loxP-*flanked gene segments (*EGFP* *3xSTOP*) in all cell types (30). BMSCs isolated from either the unrecombined (*p16-LOX-ATTAC*) or recombined (*CMV-Cre^+/–^* *p16-LOX-ATTAC*) mice were then placed in culture and irradiated (10 Gy) to induce in vitro senescence, which resulted in robust *EGFP* mRNA expression driven by the *p16^Ink4a^* promoter in unrecombined Sn BMSCs (Supplemental Figure 2C). In contrast, *EGFP* transcript levels were not detected (all cycle threshold [Ct] values = 40) in Sn BMSCs from *CMV-Cre^+/–^* *p16-LOX-ATTAC* mice (Supplemental Figure 2C), thus confirming *EGFP* excision. Consistent with this, IHC staining of FLAG, which tags the FKBP-Casp8 portion of the construct, revealed that the FKBP-Casp8 protein was not expressed in Sn BMSCs from *p16-LOX-ATTAC* mice unless the cells were derived from *p16-LOX-ATTAC* mice crossed with a *Cre* (e.g., *CMV-Cre*; Supplemental Figure 2D).

To validate this model in vivo, we crossed *p16-LOX-ATTAC* mice with *E2a-Cre* mice, which targets expression of *Cre* to the early mouse embryo, thus causing *Cre*-mediated recombination in a wide range of tissues (31). We next used a well-established drug-inducible model (i.e., doxorubicin [DoxR]) of cellular senescence in vivo (32, 33). We randomized 4-month-old young adult *E2a-Cre*^+/–^ *p16-LOX-ATTAC* mice to 1 of 3 groups: (a) vehicle (Veh); (b) DoxR (10 mg/kg) plus Veh; or (c) DoxR (10 mg/kg) plus AP (10 mg/kg, twice weekly) for 24 days (Supplemental Figure 2E). As an indicator of senescence induction, DoxR treatment increased *p16^Ink4a^* mRNA expression in liver tissue, whereas AP was sufficient to counteract this effect (Supplemental Figure 2F), thus establishing in vivo functionality.

### Specific elimination of Sn osteocytes in old mice.

To specifically eliminate Sn osteocytes in old age, we crossed *p16-LOX-ATTAC* mice with *8kb* *DMP1-Cre* mice (14) and aged the female and male *DMP1-Cre^+/–^* *p16-LOX-ATTAC* mice to 20 months (baseline). The mice were then randomized to either Veh or AP (10 mg/kg) twice-weekly i.p. injections for 4 months until 24 months of age (Figure 1G). Compared with Veh, 4 months of AP treatment significantly reduced *p16^Ink4a^* mRNA expression in osteocyte-enriched bones of both female and male *DMP1-Cre^+/–^* *p16-LOX-ATTAC* mice but had no effect on *p16^Ink4a^* transcript levels in other tissues (Figure 1H), indicating efficient osteocyte-specific senolysis. Results were similar in both females and males when analyzed separately (Supplemental Figure 3, A and B). Osteocytes from *DMP1-Cre^+/–^* *p16-LOX-ATTAC* mice treated with AP for 4 months had significantly lower TAF levels compared with Veh-treated mice (Figure 1I), providing confirmation of Sn osteocyte clearance. Further analyses revealed no significant difference in osteoblast TAF levels in AP- versus Veh-treated mice (Figure 1J). However, given that the *8kbDmp-1-Cre* can be active in late osteoblasts (34) and the similar (albeit nonsignificant in osteoblasts) pattern of TAF reductions in osteocytes and osteoblasts, we cannot fully exclude some effect of our interventions in clearing not only Sn osteocytes but also potentially Sn late osteoblasts. Finally, staining for senescence-associated β-galactosidase (SA–β-Gal) adipocytes in *DMP1-Cre^+/–^* *p16-LOX-ATTAC* mice revealed no SnC clearance in adipose tissue (Figure 1, K and L), thus providing further evidence that only Sn osteocytes were eliminated in response to AP treatment.

### Effects of osteocyte senolysis on body composition and the skeleton in old mice.

**Figure 2:**
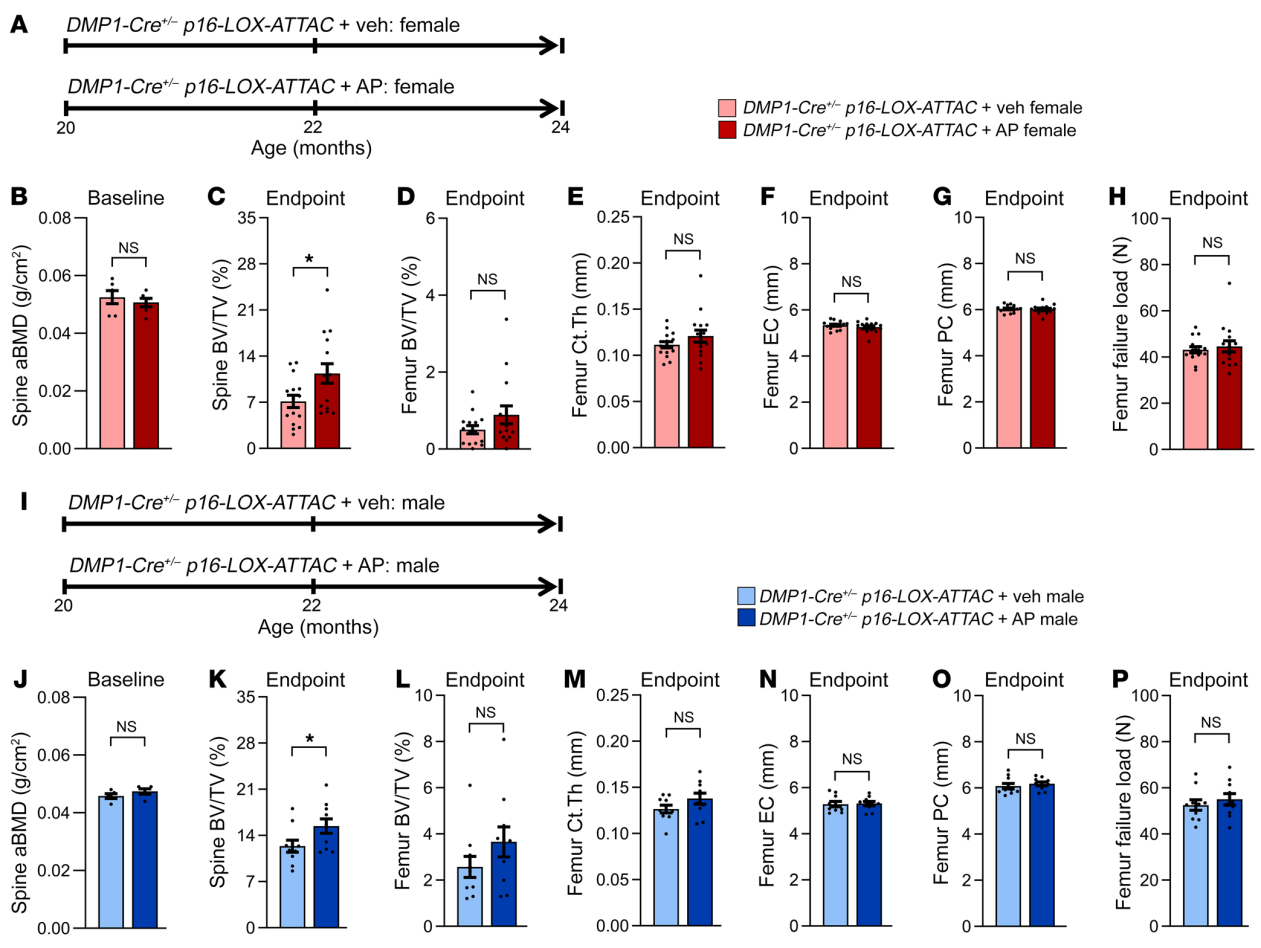
Effects of local Sn osteocyte–specific clearance on the skeleton of old female and male mice. (**A**) Study design for local clearance of Sn osteocytes in female old (20 months) *DMP1-Cre*^+/–^ *p16-LOX-ATTAC* mice randomized to Veh (pink) or AP (red) treatment for 4 months. (**B**) DXA-derived aBMD (g/cm^2^) at baseline in 20-month-old females (*n* = 6 females/group). (**C**) Quantification of the study endpoint (24 months) μCT-derived bone volume BV/TV fraction at the lumbar spine in mice treated with Veh (*n* = 15 females) versus AP (*n* = 15 females). (**D**–**H**) Quantification of μCT-derived (**D**) BV/TV, (**E**) cortical thickness (Ct.Th), (**F**) endocortical circumference (EC), (**G**) periosteal circumference (PC), and (**H**) μFEA-derived failure load at the femur metaphysis in female mice (*n* = 15 females/group). (**I**) Study design for local clearance of Sn osteocytes in old (20 months) male *DMP1-Cre*^+/–^ *p16-LOX-ATTAC* mice randomized to Veh (light blue) or AP (dark blue) treatment for 4 months. (**J**) DXA-derived aBMD (g/cm^2^) at baseline in 20-month-old male mice (*n* = 5/males group). (**K**) Quantification of the study endpoint (24 months) μCT-derived BV/TV at the lumbar spine in male mice treated with Veh (*n* = 10 males) or AP (*n* = 10 males). (**L**–**P**) Quantification of μCT-derived (**L**) BV/TV, (**M**) cortical thickness, (**N**) endocortical circumference, (**O**) periosteal circumference, and (**P**) μFEA-derived failure load at the femur metaphysis in male mice (*n* = 10/group). Data represent the mean ± SEM. NS, *P* > 0.05; *P < 0.05 and **P < 0.01, by independent samples Student’s *t* test or Wilcoxon rank-sum test, as appropriate.

To assess the effect of clearing Sn osteocytes on body composition and age-related bone loss, we performed echomagnetic resonance imaging (echo-MRI), dual-energy x-ray absorptiometry (DXA), micro-CT (μCT), and measurements of bone histomorphometry. As noted above, AP or Veh was delivered i.p. to *DMP1-Cre^+/–^* *p16-LOX-ATTAC* female and male mice from 20–24 months of age (Figure 2, A and I). Mice that received AP exhibited no changes in body weight at baseline or monthly thereafter when compared with Veh-treated mice (Supplemental Figure 4, A–D). Similarly, total body fat mass (Supplemental Figure 4, E and F) and lean mass (Supplemental Figure 4, G and H) did not differ between AP- and Veh-treated mice at baseline (20 months) or at the study endpoint (24 months). Furthermore, we observed no effects of AP versus Veh on any body composition parameters when females (Supplemental Figure 5, A–H) and males (Supplemental Figure 6, A–H) were analyzed separately.

Note that for the primary study endpoints (μCT outcomes in the different mouse models), the data are shown separately for females and males (including both the main and supplemental figures). In addition, for each of the μCT outcomes when combining males and females, we performed ANOVA statistical models testing for a sex-by-treatment (sex × treatment) interaction, as recommended by Garcia-Sifuentes and Maney (35) (all *P* > 0.05, Supplemental Table 1). Female mice were randomized to either Veh or AP (10 mg/kg) twice-weekly i.p. injections for 4 months until 24 months of age (Figure 2A). AP- and Veh-treated female mice were well matched at baseline (20 months) for lumbar spine DXA-derived areal bone mineral density (aBMD) (Figure 2B). Compared with Veh, Sn osteocyte–specific clearance in response to 4 months of AP treatment in old female mice significantly improved the lumbar spine trabecular bone volume fraction (BV/TV) (Figure 2C). By contrast, clearance of Sn osteocytes in female mice had no effect on femur trabecular BV/TV (Figure 2D), femur cortical bone parameters (Figure 2, E–G), or bone strength (failure load, assessed by microfinite element analysis [μFEA]) (Figure 2H). Consistent with the results in female mice, AP- and Veh-treated male mice (Figure 2I) were well matched at baseline (20 months) for lumbar spine DXA-derived aBMD (Figure 2J). Furthermore, compared with Veh, Sn osteocyte–specific clearance in response to 4 months of AP treatment in old male mice significantly improved lumbar spine trabecular BV/TV (Figure 2K), whereas clearance of Sn osteocytes in male mice had no effect on femur trabecular BV/TV (Figure 2L), femur cortical bone parameters (Figure 2, M–O), or bone strength (failure load, assessed by μFEA) (Figure 2P).

**Figure 3:**
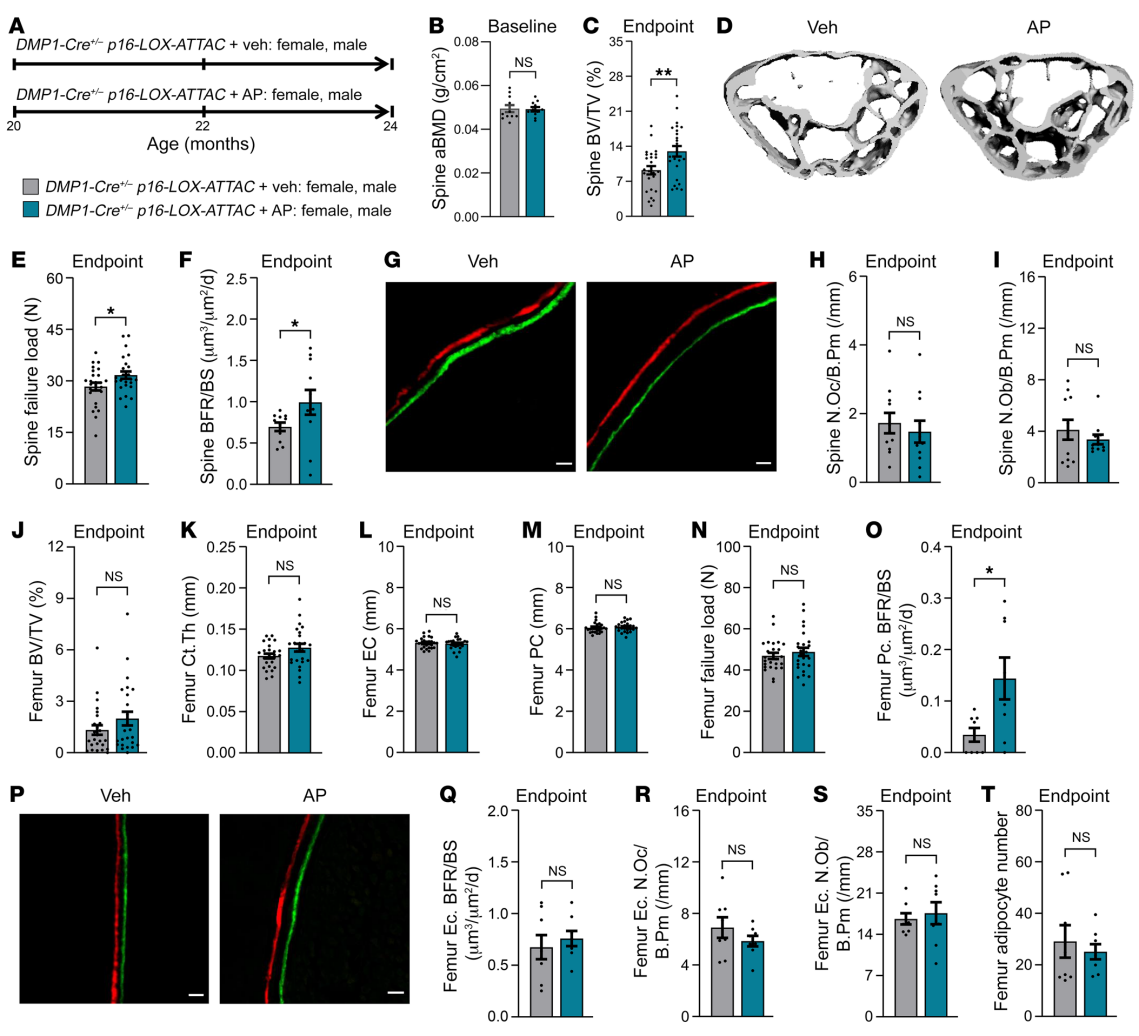
Effects of local Sn osteocyte–specific clearance on the skeleton of old mice. (**A**) Study design in old (20 months) *DMP1-Cre*^+/–^ *p16-LOX-ATTAC* mice, males and females combined, randomized to Veh (gray) or AP (teal). (**B**) DXA-derived aBMD (g/cm^2^) at baseline (20 months; *n* = 11/group: *n* = 6 females, *n* = 5 males per group). (**C**) Endpoint lumbar spine μCT-derived bone volume fraction (BV/TV; %) in Veh-treated (*n* = 25; *n* = 15 females, *n* = 10 males) versus AP-treated (*n* = 25: *n* = 15 females, *n* = 10 males) mice. (**D**) Representative spine μCT images of Veh- versus AP-treated mice. (**E**) Lumbar spine μFEA-derived failure load in Veh-treated mice (*n* = 25: *n* = 15 females, *n* = 10 males) versus AP-treated (*n* = 25: *n* = 15 females, *n* = 10 males). (**F**) Lumbar spine BFR per bone surface (BFR/BS) (μm^3^/μm^2^/d; *n* = 11/group: *n* = 6 females, *n* = 5 males per group). (**G**) Lumbar spine representative images of Alizarin red and calcein fluorochrome dynamic BFR labels on trabecular surfaces in Veh- and AP-treated mice. Scale bars: 10 μm. (**H** and **I**) Lumbar spine histomorphometric quantification (*n* = 8/group: *n* = 4 females, *n* = 4 males per group) of (**H**) osteoclast numbers per bone perimeter (N.Oc/B.Pm/mm) and (**I**) osteoblast numbers per bone perimeter (N.Ob/B.Pm/mm). (**J**–**N**) Femur metaphysis μCT-derived (**J**) BV/TV, (**K**) cortical thickness, (**L**) endocortical circumference, (**M**) periosteal circumference, and (**N**) μFEA-derived failure load (*n* = 25/group: *n* = 15 females, *n* = 10 males per group). (**O**) Femur histomorphometric quantification (*n* = 8/group: *n* = 4 females, *n* = 4 males per group) of periosteal cortical (Pc.) BFR/BS (μm^3^/μm^2^/d). (**P**) Representative images of Alizarin red and calcein fluorochrome dynamic BFR labeling on periosteal cortical surfaces of femurs from Veh- and AP-treated mice. Scale bars: 10 μm. (**Q**–**T**) Femur histomorphometric quantification (*n* = 8/group: *n* = 4 females, *n* = 4 males per group) of (**Q**) endocortical (Ec.) BFR/BS (μm^3^/μm^2^/d), (**R**) endocortical N.Oc/B.Pm (/mm), (**S**) endocortical N.Ob/B.Pm (/mm), and (**T**) bone marrow adipocyte numbers. Data represent the mean ± SEM. NS, *P* > 0.05; *P < 0.05 and **P < 0.01, by independent samples Student’s *t* test or Wilcoxon rank-sum test, as appropriate.

When female and male mice were combined (Figure 3A), following ANOVA testing for sex × treatment interactions (all *P* > 0.05, Supplemental Table 1), AP- and Veh-treated mice were again well matched at baseline (20 months) for lumbar spine DXA-derived aBMD (Figure 3B). Compared with Veh, Sn osteocyte–specific clearance in response to 4 months of AP treatment significantly improved the lumbar spine BV/TV fraction (Figure 3, C and D) and bone strength (failure load, assessed by μFEA) (Figure 3E) by significantly increasing bone formation rates (BFRs) on spine trabecular surfaces (Figure 3, F and G). However, clearance of Sn osteocytes in AP-treated mice had no effect on spine osteoclast (Figure 3H) or osteoblast (Figure 3I) numbers. The increase in the spine BFR without an increase in osteoblast numbers would indicate an increase in osteoblast activity accounting for the increase in the BFR. We also measured the serum bone formation marker amino-terminal propeptide of type 1 collagen (P1NP), which did not differ between groups (Veh, 1.39 ± 0.36 vs. AP, 1.62 ± 0.19 ng/mL, *P* = 0.352), likely due to insufficient sensitivity of this marker to detect changes in the trabecular BFR in the absence of changes in the endocortical BFR along the much more prevalent cortical bone (see below). At the femur metaphysis, Sn osteocyte clearance in AP-treated mice did not affect femur BV/TV (Figure 3J) and only resulted in a modest, albeit nonsignificant (*P* = 0.078), increase in cortical thickness (Figure 3K) without affecting endocortical (Figure 3L) or periosteal (Figure 3M) circumferences. Collectively, 4 months of AP treatment in old *DMP1-Cre^+/–^* *p16-LOX-ATTAC* mice was not sufficient to significantly change femoral bone strength (μFEA-derived failure load; Figure 3N). However, Sn osteocyte clearance in AP-treated mice did significantly improve BFRs on periosteal (Figure 3, O and P), but not endocortical (Figure 3Q), surfaces of the femur metaphysis, thus explaining the modest improvement in cortical thickness observed in AP-treated mice. Clearance of Sn osteocytes in AP-treated mice had no effect on femoral osteoclast (Figure 3R), osteoblast (Figure 3S), or marrow adipocyte (Figure 3T) numbers.

In addition to aging the *DMP1-Cre^+/–^* *p16-LOX-ATTAC* mouse colony, as a control we also aged a separate cohort of *p16-LOX-ATTAC* mice not crossed with a *Cre* (noncrossed controls) to 20 months of age and delivered AP or Veh i.p. (10 mg/kg, twice weekly) to females and males for 4 months (Supplemental Figures 7–9). Aged female *p16-LOX-ATTAC* (noncrossed) mice did not differ at baseline (20 months) for spine aBMD (Supplemental Figure 7B). Furthermore, after 4 months of Veh or AP treatment, we found no differences in spine trabecular parameters (Supplemental Figure 7, C–F), femur trabecular BV/TV (Supplemental Figure 7G), femur cortical parameters (Supplemental Figure 7, H–K), or femoral bone strength (μFEA-derived failure load; Supplemental Figure 7L) between old female *p16-LOX-ATTAC* (noncrossed) Veh- and AP-treated mice. The results were similar in aged male *p16-LOX-ATTAC* (noncrossed) Veh- and AP-treated mice, in which no between-groups differences in bone parameters were observed (Supplemental Figure 8, A–L). In addition when combining sexes, following ANOVA testing for sex × treatment interactions (Supplemental Table 1), both groups were well matched at baseline (20 months) for spine aBMD (Supplemental Figure 9B), and 4 months of AP treatment had no significant effects on μCT-derived bone parameters at the lumbar spine (Supplemental Figure 9, C–F) or femur metaphysis (Supplemental Figure 9, G–L), thus demonstrating that the *ATTAC* portion of the *p16-LOX-ATTAC* transgene is not activated by AP if these mice are not crossed with a *Cre* model (i.e., there is no “leakiness” of the *p16-LOX-ATTAC* transgene).

Finally, we aged another separate cohort of *p16-LOX-ATTAC* mice crossed with *β-actin–Cre* mice, which results in widespread tissue targeting of *Cre* (36), and we aged the resulting *β-actin–Cre^+/–^* *p16-LOX-ATTAC* mice to 20 months, at which point AP or Veh was delivered i.p. (10 mg/kg, twice weekly) to females and males for 4 months (Supplemental Figure 10A). Consistent with previously published findings in response to systemic SnC clearance in old *p16-INK-ATTAC* mice (10, 27, 37), AP treatment in old *β-actin–Cre^+/–^* *p16-LOX-ATTAC* mice resulted in significantly reduced *p16^Ink4a^* mRNA expression in multiple tissues, including osteocyte-enriched bones, fat, kidney, and liver (Supplemental Figure 10B). Similar effects were observed in females and males when analyzed separately, although because of the variability of the *p16^Ink4a^* mRNA measurements, some differences between the sexes were evident, albeit not significant (all *P* > 0.05) for a sex × treatment interaction by ANOVA (Supplemental Figure 10, C and D, and Supplemental Table 1).

Consistent with Sn osteocyte–specific clearance, systemic SnC clearance in old male *β-actin–Cre^+/–^* *p16-LOX-ATTAC* mice had no significant effects on body weight (Supplemental Figure 11, A–D), fat mass (Supplemental Figure 11, E–G), or lean mass (Supplemental Figure 11, H–J), and while both male groups were well matched at baseline for spine aBMD (Supplemental Figure 11K), 4 months of AP treatment as compared with Veh in old male mice resulted in significant improvements in lumbar spine BV/TV (Supplemental Figure 11L), femur BV/TV (Supplemental Figure 11M), femur cortical thickness (Supplemental Figure 11N), and femoral bone strength (μFEA-derived failure load, Supplemental Figure 11O). In old female *β-actin–Cre^+/–^* *p16-LOX-ATTAC* mice, the results were similar (Supplemental Figure 12, A–O); however, improvements in response to AP treatment did not reach statistical significance (i.e., using parametric statistics) for trabecular BV/TV at the spine (Supplemental Figure 12L; Student’s *t* test *P* = 0.070; Wilcoxon rank-sum test *P* = 0.065) or trabecular BV/TV at the femur (Supplemental Figure 12M; Student’s *t* test *P* = 0.138; Wilcoxon rank-sum test *P* = 0.034) because of the suboptimal statistical power as a result of higher-than-anticipated numbers of deaths in the aged *β-actin–Cre^+/–^* *p16-LOX-ATTAC* cohort. Notwithstanding, despite the relatively smaller sample size (*n* = 11 Veh-treated females vs. *n* = 11 AP-treated females) compared with our previously published study in aged female *p16-INK-ATTAC* mice (*n* = 13 Veh-treated vs. *n* = 16 AP-treated), in which systemic clearance of SnCs significantly improved trabecular bone parameters at both the spine and femur as well as femur cortical bone parameters (27), in the present study, old female *β-actin–Cre^+/–^* *p16-LOX-ATTAC* mice treated with AP had significant improvements in femur cortical thickness (Supplemental Figure 12N) and femoral strength (failure load assessed by μFEA) (Supplemental Figure 12O). When combining the aged female and male *β-actin–Cre^+/–^* *p16-LOX-ATTAC* mice, following ANOVA testing for sex × treatment interactions (Supplemental Table 1), we found that AP- and Veh-treated mice had similar body compositions (Supplemental Figure 13, A–J) and were well matched at baseline for spine aBMD (Supplemental Figure 13K), whereas 4 months of AP treatment as compared with Veh resulted in significant improvements in lumbar spine BV/TV (Supplemental Figure 13L), femur BV/TV (Supplemental Figure 13M), femur cortical thickness (Supplemental Figure 13N), and femoral bone strength (μFEA-derived failure load, Supplemental Figure 13O). Taken together, these results demonstrate that although some effects of Sn osteocyte–specific clearance on the skeleton were consistent with systemic SnC clearance (e.g., BV/TV at the spine), other effects were quite different than those observed in old *p16-INK-ATTAC* mice, as previously reported by our group (27).

### Effects of osteocyte senolysis on the osteocyte network.

**Figure 4:**
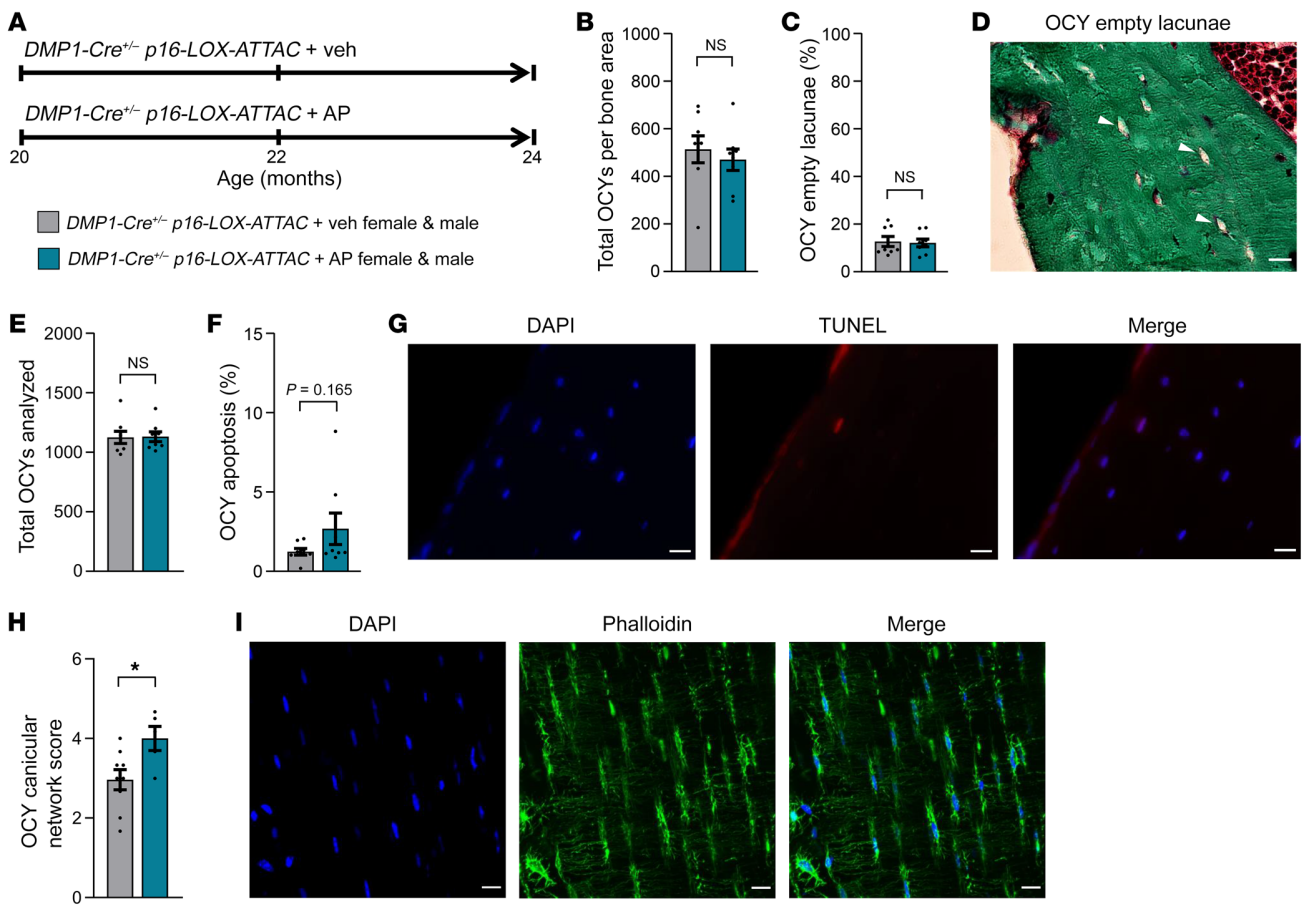
Effects of local Sn osteocyte clearance on empty lacunae, osteocyte apoptosis, and osteocyte LCN quality. (**A**) Study design for the local clearance of Sn osteocytes in old (20 months) *DMP1-Cre^+/–^* *p16-LOX-ATTAC* mouse cohorts, males and females combined, randomized to Veh (gray) or AP (teal) treatment for 4 months. (**B**–**D**) Study endpoint (24 months) quantification of (**B**) total osteocytes per bone area and (**C**) percentage of osteocyte empty lacunae in mice treated with Veh (*n* = 8: *n* = 4 females, *n* = 4 males) versus AP (*n* = 8: *n* = 4 females, *n* = 4 males), and (**D**) representative images of osteocyte empty lacunae (arrowheads). Scale bar: 25 μm. (**E**–**G**) Quantification of osteocyte apoptosis, including (**E**) total numbers of osteocytes analyzed and (**F**) percentage of osteocyte apoptosis in mice treated with Veh (*n* = 8: *n* = 4 females, *n* = 4 males) versus AP (*n* = 8: *n* = 4 females, *n* = 4 males), and (**G**) representative images of DAPI-stained, TUNEL^+^, and merged apoptotic osteocytes. Scale bars: 25 μm. (**H**) Quantification of osteocyte LCN score for mice treated with Veh (*n* = 9: *n* = 5 females, *n* = 4 males) versus AP (*n* = 5: *n* = 3 females, *n* = 2 males), and (**I**) representative images of DAPI-stained, phalloidin-stained, and merged osteocyte LCNs. Scale bars: 25 μm. Data represent the mean ± SEM. NS, *P* > 0.05; *P < 0.05, by independent samples Student’s *t* test or Wilcoxon rank-sum test, as appropriate.

Because aging causes detrimental changes to the osteocyte lacunocanalicular network (LCN), we next examined the impact of Sn osteocyte–specific clearance on multiple aspects of the osteocyte LCN in old *DMP1-Cre^+/–^* *p16-LOX-ATTAC* mice treated with AP versus Veh for 4 months from 20–24 months of age (Figure 4A). Examination of more than 400 osteocytes per mouse (Figure 4B) revealed that there were no changes in the percentage of osteocyte empty lacunae between the AP- and Veh-treated groups (Figure 4, C and D). Additional analysis of more than 1,000 osteocytes per animal (Figure 4E) suggested a modest increase in the percentage of osteocyte apoptosis as assessed by TUNEL assay in the AP- versus Veh-treated mice (Figure 4, F and G), although this increase did not reach statistical significance (*P* = 0.165, Wilcoxon nonparametric test). Interestingly, although the quality of the osteocyte LCN declined, as reflected by the number of osteocyte dendrites and their density, which both naturally degenerate in mice with aging (38), 4 months of AP treatment compared with Veh treatment in old *DMP1-Cre^+/–^* *p16-LOX-ATTAC* mice was sufficient to significantly improve the quality of the osteocyte LCN (Figure 4H; see Methods for a detailed description of the osteocyte LCN score), as assessed by phalloidin staining and confocal imaging (Figure 4I).

### Effects of local versus systemic senolysis on the SASP and senescence-related pathways.

In order to compare changes in the bone SASP as well as senescence-related genes and to characterize the key underlying intercellular signaling pathways in response to Sn osteocyte–specific (local) versus systemic SnC clearance, we next used a gene set (SenMayo) that has been previously validated for identifying SnCs across multiple tissues and species, including mice and humans, with high fidelity (39). SenMayo is a senescence gene set composed of 117 genes in mice that consists predominantly of SASP factors (*n* = 76) but also includes transmembrane (*n* = 19) and intracellular (*n* = 22) proteins. In our previous analysis of young versus old mice, we found that SenMayo expression increased with aging and that systemic SnC clearance (using *p16-INK-ATTAC* mice) following 4 months of AP treatment reduced the expression of multiple SASP- and senescence-related genes (39).

**Figure 5:**
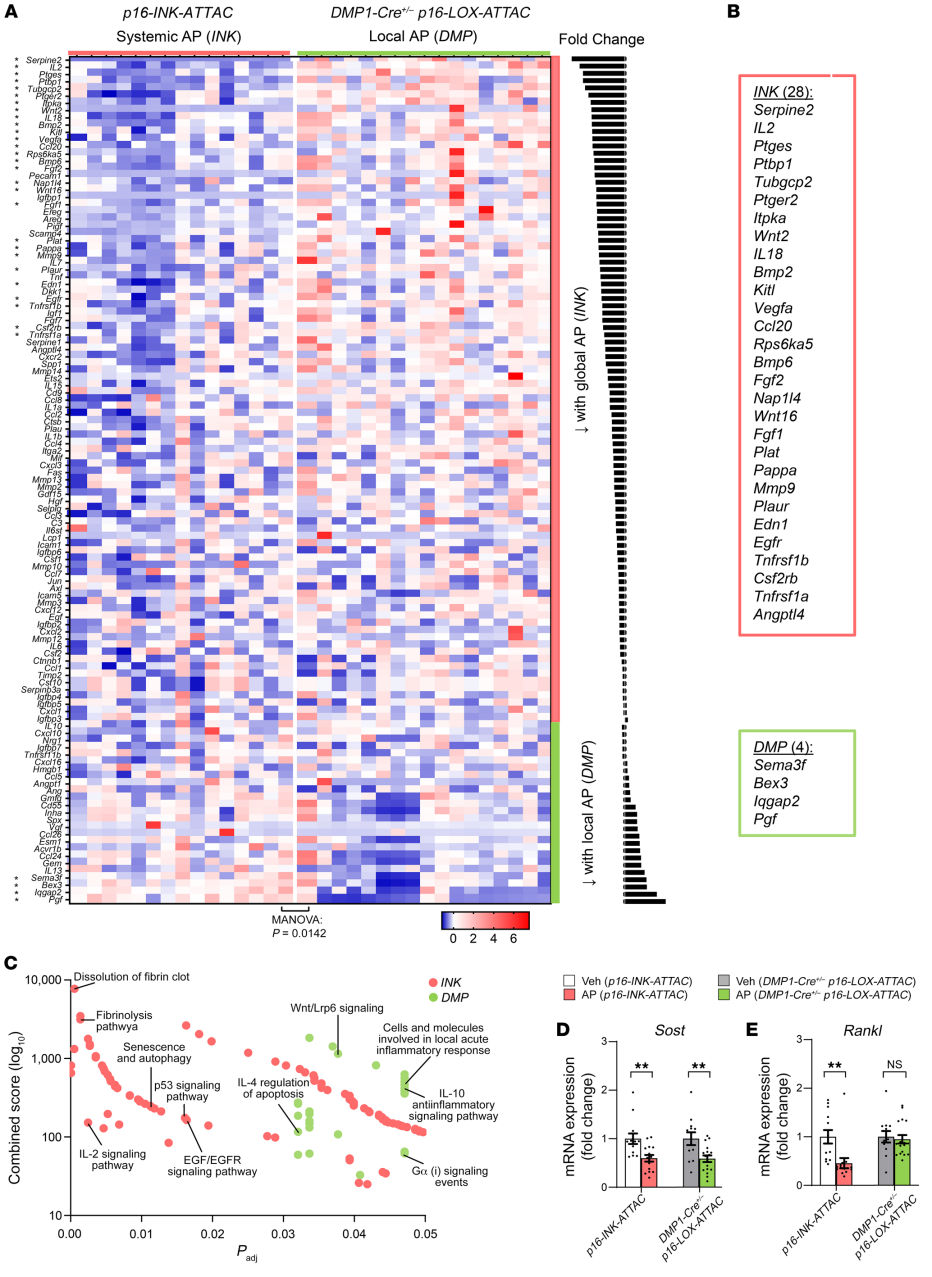
Changes in SenMayo genes and regulators of bone remodeling in response to local osteocyte-specific versus systemic SnC clearance. (**A** and **B**) After 2 weeks, a comparison of the 2 AP-treated groups (*INK* versus *DMP*) revealed broader downregulation of SASP- and Sn-related genes in the systemic SnC clearance model (*INK*) versus the local osteocyte-specific model (*DMP*). (**C**) BioPlanet 2019 pathway analysis emphasized the senescence and autophagy as well as the p53/Il2/Egf pathways as being regulated in the *INK* model, whereas the Wnt/Lrp6/IL10/IL4 pathways were regulated in the *DMP* model. (**D** and **E**) Changes in mRNA expression of (**D**) *Sost* (encoding sclerostin) and (**E**) *Rankl* (also known as *Tnfsf11*) in response to systemic (*INK*) versus local osteocyte-specific (*DMP*) senolysis (*INK* Veh *n* = 15, *INK* AP *n* = 15; *DMP* Veh *n* = 15, *DMP* AP *n* = 15). NS, *P* > 0.05; **P < 0.01, by independent samples Student’s *t* test or Wilcoxon rank-sum test, as appropriate.

Here, we used SenMayo to examine earlier changes in the SASP- and senescence-related transcriptome (after only 2 weeks of AP) in the bones of old mice following Sn osteocyte–specific (*DMP1-Cre^+/–^* *p16-LOX-ATTAC*) or systemic (*p16-INK-ATTAC*) SnC clearance. Application of SenMayo to whole bones (i.e., not specifically enriched for osteocytes) obtained from 24-month-old *DMP1-Cre^+/–^* *p16-LOX-ATTAC* and *p16-INK-ATTAC* mice showed highly significant downregulation of the SenMayo gene set by AP treatment in both *p16-INK-ATTAC* and *DMP1-Cre^+/–^* *p16-LOX-ATTAC* mice (Supplemental Figure 14; multivariate analysis of variance [MANOVA] for overall comparison of gene sets, *P* < 0.005 for both [see *Statistics*]), consistent with a senolytic effect of AP in both models. Next, to directly interrogate genes differentially altered by senolysis in the osteocyte-specific (local) versus systemic SnC clearance models, we compared the SenMayo panel between the AP-treated groups. This comparison revealed a large majority of genes that were lower in the systemic model compared with the local model (Figure 5A), consistent with a more profound senolytic effect in response to systemic SnC clearance. Specifically, when considering the individual SenMayo genes that were significantly different between the AP-treated groups, systemic senolysis resulted in 28 genes that were significantly lower relative to local senolysis, whereas local senolysis resulted in only 4 genes that were significantly lower when compared with systemic senolysis (Figure 5B). We next applied BioPlanet 2019 pathway analysis (Figure 5C), which revealed the senescence and autophagy as well as the p53/IL2/Egf/Egfr pathways as being the most regulated after systemic senolysis and the Wnt/Lrp6/IL10/IL4 pathways as the most regulated following local senolysis.

To further extend these findings, we next measured an array of cytokines and chemokines (total of 32 protein targets) in serum from these old mice. With local senolysis, we found that only a single target (IP10) was reduced, whereas 10 targets (GM-CSF, IFN-γ, IL-13, IL-17, MCP-1, MIP1A, MIP2B, RANTES, TNF-α, and VEGF) were reduced in response to systemic senolysis, although when considering the entire array using MANOVA, between-groups differences were not significant following Sn osteocyte–specific (*DMP1-Cre^+/–^* *p16-LOX-ATTAC*; MANOVA *P* = 0.247) or systemic (*p16-INK-ATTAC*; MANOVA *P* = 0.509) SnC clearance (Supplemental Figure 15, A and B). Importantly, however, when the array of cytokines and chemokines was restricted to only those targets included in our recently validated senescence gene set (SenMayo; ref. 39), whereas changes in these targets remained nonsignificant (MANOVA *P* = 0.744) following local senolysis (Supplemental Figure 16A), systemic senolysis resulted in a significant (MANOVA *P* = 0.045) reduction in SenMayo proteins, including IL-13, MCP-1, MIP1A, MIP1B, RANTES, TNF-α, and VEGF (Supplemental Figure 16B). Collectively, these data indicate that systemic senolysis has greater effects on circulating inflammatory cytokines as compared with osteocyte-specific senolysis and, moreover, that the reduction in inflammatory factors following systemic senolysis is relatively specific for known SASP factors.

Finally, in addition to overlap as well as differences in SASP- and senescence-related genes and pathways, further RT-qPCR analyses revealed that *Sost* (encoding sclerostin) levels in whole bones were significantly reduced with both systemic and local senolysis (Figure 5D), whereas only with systemic senolysis, but not local senolysis, was *Rankl* (also known as *Tnfsf11*) significantly reduced in whole bones of old AP- relative to Veh-treated mice (Figure 5E). These findings are entirely consistent with the increase in bone formation observed in both models, but a decrease in bone resorption that occurred only in the systemic senolysis model.

### Transplanting SnCs into young mice leads to skeletal aging.

**Figure 6:**
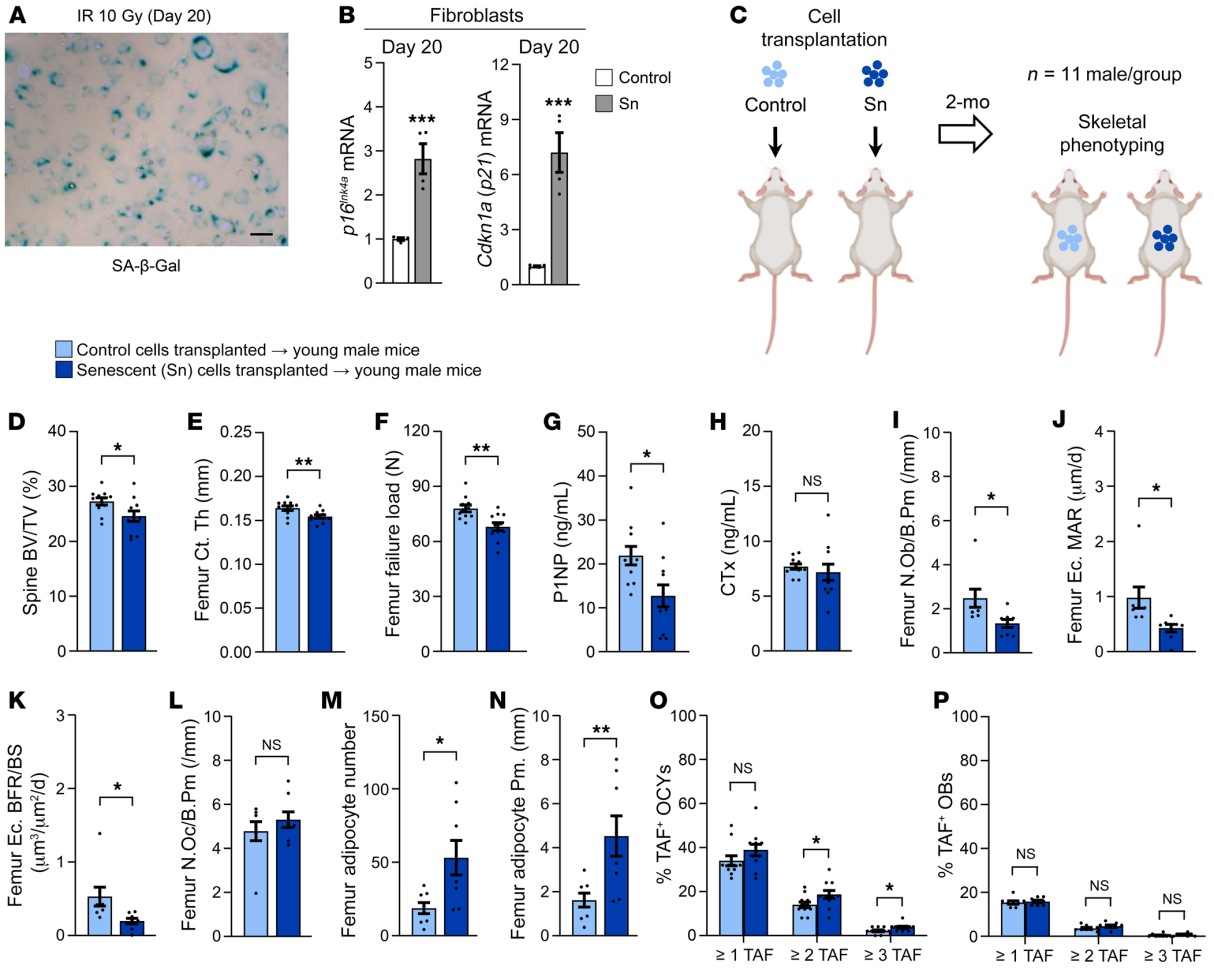
Transplantation of cells made Sn by IR causes skeletal aging in young adult mice. (**A**) Confirmation of cellular senescence 20 days after 10 Gy IR using SA–β-Gal staining. Scale bar: 25 μm. (**B**) RT-qPCR analysis of *p16^Ink4a^* and *p21^Cip1^* mRNA expression in Sn versus control fibroblasts 20 days after 10 Gy IR. (**C**) Study design for control and Sn cell transplantation (via i.p. injection) and skeletal phenotyping in young adult male *C57BL/6* WT mice (*n* = 11 males/group). (**D**–**F**) Quantification of μCT-derived (**D**) lumbar spine BV/TV fraction, (**E**) femur metaphysis cortical thickness, and (**F**) femur metaphysis μFEA-derived failure load (i.e., bone strength) in mice transplanted with control (*n* = 11 males) versus Sn (*n* = 11 males) cells. (**G** and **H**) Quantification of circulating serum bone turnover markers, including (**G**) the bone formation marker P1NP (ng/mL) and (**H**) the bone resorption marker cross-linked CTx (ng/mL) (*n* = 11 males/group). (**I**–**L**) Histomorphometric quantification at the femoral endocortical surface of (**I**) osteoblast numbers per bone perimeter (/mm), (**J**) mineral apposition rate (MAR) (μm/d), (**K**) BFR/BS (μm^3^/μm^2^/d), and (**L**) osteoclast numbers per bone perimeter (/mm) (*n* = 8 males/group). (**M** and **N**) Histomorphometric quantification of femur bone marrow (**M**) adipocyte numbers and (**N**) adipocyte perimeter (mm) (*n* = 8 males/group). (**O** and **P**) Quantification of (**P**) the mean percentage of TAF^+^ osteocytes per mouse and (**P**) the mean percentage of TAF^+^ osteoblasts per mouse based on the following criteria: the percentage of cells with 1 or more TAF, the percentage of cells with 2 or more TAF, and the percentage of cells with 3 or more TAF, respectively. Data represent the mean ± SEM. NS, *P* > 0.05; *P < 0.05, **P < 0.01, and ***P < 0.001, by independent samples Student’s *t* test or Wilcoxon rank-sum test, as appropriate.

To test whether nonskeletal SnCs can induce aging bone phenotypes, we first developed a reproducible protocol for isolating primary fibroblasts from young adult C57BL/6 mice and making them Sn in culture. Indeed, in vitro exposure of primary murine fibroblasts to 10 Gy irradiation (IR), followed by a 20-day waiting period, caused most surviving IR-exposed cells (relative to non-IR, control cells) to become Sn using criteria based on the combination of SA–β-Gal^+^ staining and markedly increased mRNA expression of both *p16^Ink4a^* and *p21^Cip1^* (Figure 6, A and B). We previously found that the SASP of IR-induced Sn murine fibroblasts is consistent with that of in vivo SnCs with aging (40, 41) and, using luciferase tracking, that these transplanted SnCs remain in the peritoneal cavity following transplantation by i.p. injection and do not travel to distant organs including bone (42).

We next transplanted Sn or non-Sn (control) murine primary fibroblasts (~10^6^) isolated from young adult C57BL/6 WT mice into young adult (4-month-old), syngeneic male WT mice and then waited 2 months before sacrificing these animals, subsequently performing extensive skeletal phenotyping (Figure 6C). At 6 months of age, μCT of the lumbar spine revealed that mice transplanted with approximately 10^6^ SnCs versus similar numbers of non-Sn control cells had significantly lower trabecular BV/TV (Figure 6D). Similarly, SnC transplantation caused cortical thinning and deficient bone strength (i.e., μFEA-derived failure load) at the femur metaphysis (Figure 6, E and F). SnC transplantation significantly reduced circulating serum levels of the bone formation marker P1NP in young mice transplanted with SnCs versus non-Sn control cells (Figure 6G) but had no effect on circulating levels of the bone resorption marker serum C-terminal telopeptide of type I collagen (CTx) (Figure 6H). Consistent with these findings in the circulation, bone histomorphometry revealed that both osteoblast numbers (Figure 6I) and BFRs (Figure 6, J and K) were significantly reduced in young mice transplanted with SnCs, but there were no changes in osteoclast numbers in SnC-transplanted compared with non-Sn control cell–transplanted young mice (Figure 6L). In contrast with the deficient numbers of osteoblasts, we observed a marked increase in marrow adipocytes in SnC-transplanted mice compared with young mice transplanted with non-Sn control cells (Figure 6, M and N). Finally, because our group has previously shown that transplanted cells (both Sn and non-Sn control) remain detectable in vivo for only up to 40 days after transplantation (42), we next asked whether at 60 days after SnC transplantation, beyond the point these cells are no longer detectable, the transplanted SnCs induced senescence in normal, previously healthy host cells in vivo. Indeed, 2 months after transplantation, TAF staining revealed significantly more TAF^+^ osteocytes (Figure 6O), but no difference in osteoblast TAF levels (Figure 6P), in SnC-transplanted mice compared with mice transplanted with non-Sn control cells, thus demonstrating that senescence had spread to distant host osteocytes.

### The SASP impairs bone formation and alters MSC lineage commitment.

**Figure 7:**
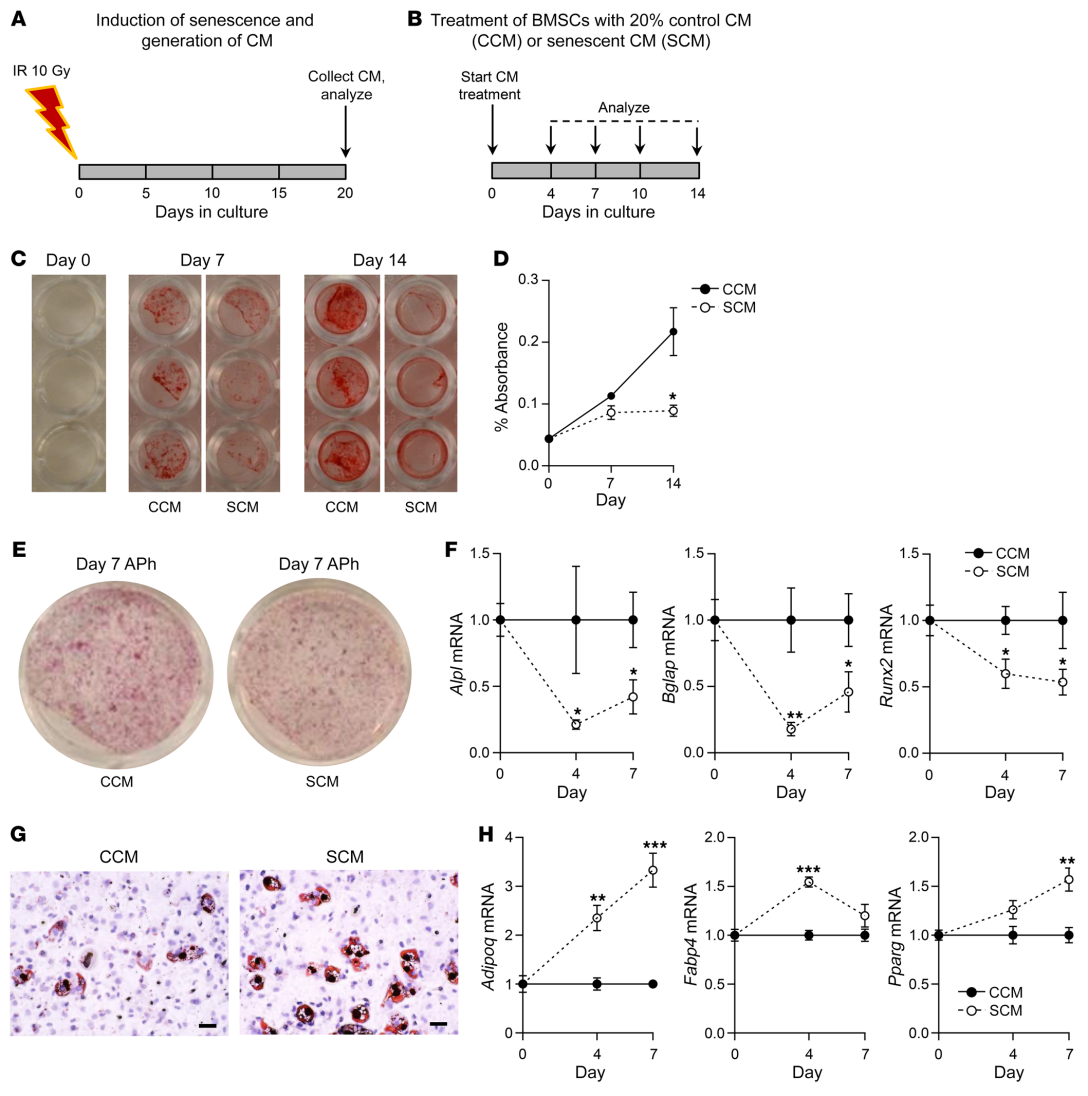
The SASP impairs bone formation and enhances bone marrow adipogenesis. (**A**) Schematic of in vitro cellular senescence induction using 10 Gy IR and generation of CM following 20 days in culture. (**B**) Study design depicting the treatment of BMSCs with 20% control CM (CCM) or Sn CM (SCM) for 14 days. (**C**) Mineralization of BMSCs exposed to control CM or Sn CM at days 0, 7 (*n* = 6/group), and 14 (*n* = 6/group). (**D**) Quantification of eluted Alizarin red dye from BMSCs exposed to control CM or Sn CM at days 0, 7 (*n* = 6/group), and 14 (*n* = 6/group). (**E**) Representative images of APh staining of BMSCs exposed to control CM or Sn CM on day 7. (**F**) Changes in mRNA expression of osteoblastic genes in BMSCs exposed to either control CM (*n* = 6) or Sn CM (*n* = 6). (**G**) Representative images of Oil Red O staining of BMSCs exposed to either control CM or Sn CM. Scale bars: 25 μm. (**H**) Changes in mRNA expression of adipogenic genes in BMSCs exposed to either control CM or Sn CM (*n* = 6/group). Data represent the mean ± SEM. *P < 0.05, **P < 0.01, and ***P < 0.001, by independent samples Student’s *t* test or Wilcoxon rank-sum test, as appropriate.

Because SnC clearance in aged mice improves bone formation and inhibits marrow adiposity, and in young mice SnC transplantation causes premature age-related effects on osteoblast and marrow adipocyte lineage commitment, we hypothesized that the SASP produced by SnCs is causal in the pathogenesis of the age-related defect in bone formation and alteration of MSC lineage commitment. To test this hypothesis experimentally, we induced in vitro senescence of murine primary fibroblasts (as above), and 20 days after IR, we collected the SnC conditioned medium (CM) (Figure 7A), which is a rich source of SASP factors (40–42). We then cultured BMSCs isolated from young adult C57BL/6 WT mice under osteogenic conditions in the presence of either Sn CM or control CM (Figure 7B). After 14 days, Sn CM markedly impaired the mineralization of BMSCs as compared with control CM (Figure 7, C and D). Consistent with this, Sn CM treatment reduced alkaline phosphatase (APh) staining in BMSCs treated with Sn CM versus control CM (Figure 7E). In addition, RT-qPCR analyses revealed significantly reduced mRNA levels of classic bone marker genes (i.e., *Alpl*, *Bglap*, *Runx2*) during osteoblastic differentiation of BMSCs exposed to Sn CM versus control CM (Figure 7F). Taken together, these data support a key mechanistic link between the SASP produced by SnCs and impaired bone formation, which occurs in vivo both with aging and in response to SnC transplantation in younger mice, and which can be rescued in old mice by both Sn osteocyte–specific and systemic SnC clearance. Further, because systemic, but not osteocyte-specific, clearance of SnCs resulted in reduced marrow adiposity, which could be induced in young mice transplanted with SnCs, we complemented these in vivo findings with in vitro studies, showing that treatment of primary murine BMSCs isolated from young C57BL/6 WT mice and cultured under adipogenic conditions in the presence of fibroblast Sn CM resulted in increased adipocyte formation as compared with control CM (Figure 7G) and was associated with increased mRNA expression of multiple adipogenic markers (i.e., *Adipoq*, *Fabp4*, *Pparg*) (Figure 7H).

## Discussion

Because multiple lines of evidence reinforce the premise that a single senolytic may not be capable of systemically clearing all SnC types (12), it has become of paramount importance to understand the local versus systemic effects of eliminating SnCs. Indeed, elucidating the cell-autonomous versus -nonautonomous roles of specific SnCs in mediating tissue dysfunction will help guide the development of senolytics (or their combinations) that most broadly alleviate age-related diseases as a group. Here, we developed and used a transgenic mouse that allowed for the inducible removal of specific types of SnCs. Our results establish that local senolysis in old age only partially replicates the benefits of systemic senolysis. These results were complemented by SnC transplantation studies demonstrating that the SASP from SnCs in the peritoneal cavity could induce senescence systemically in distant host osteocytes. Collectively, in addition to dissecting cell-autonomous versus -nonautonomous mechanisms of senescence, our studies indicate that optimizing senolytic drugs may require systemic “broad-spectrum” SnC targeting to extend healthy aging.

We had previously demonstrated that systemic SnC clearance using the *p16-INK-ATTAC* model prevents age-related bone loss in mice (27). Here, we used a mouse model (*p16-LOX-ATTAC*) to specifically clear Sn osteocytes, which resulted in substantial, but incomplete, skeletal benefits when compared with systemic SnC clearance using either the *p16-INK-ATTAC* model or *p16-LOX-ATTAC* mice crossed with a ubiquitously expressed *Cre* (*β-actin*). We believe that the “cleanest” controls for the *DMP1-Cre^+/–^* *p16-LOX-ATTAC* mice are the *p16-INK-ATTAC* mice, as in this model, systemic SnC clearance is assured because this construct is simply driven by the *p16^Ink4a^* promoter and does not depend on uniform expression of a “tissue-nonspecific” Cre. Specifically, although the *β-actin–Cre* is considered a ubiquitous Cre, it is probably not equally active across all tissues because of both the specific promoter and perhaps the integration site of the transgene. We acknowledge, however, that our choice of the *p16-INK-ATTAC* mice as the control in some experiments does raise the possibility that the minimally different transgene constructs between the *DMP1-Cre^+/–^* *p16-LOX-ATTAC* and the *p16-LOX-ATTAC* could be a potential confounder.

Although we found that the specific removal of Sn osteocytes in old mice was sufficient to prevent trabecular bone loss at the spine by improving bone formation, it did not alter femur trabecular bone parameters, only modestly enhanced cortical thickness by improving periosteal (but not endocortical) bone formation, and had no effects on bone resorption or marrow adiposity. The increased bone formation at the spine as well as at the periosteal cortex without changes in osteoblast numbers following osteocyte-specific senolysis suggests that synthetic activity (and/or longevity) of periosteal osteoblasts is regulated by Sn osteocytes perhaps via IGFBP5, a component of SenMayo (39) that has been previously shown to suppress bone formation on the periosteum but not the endosteum (43, 44). These findings in response to osteocyte-specific SnC clearance contrast with systemic SnC clearance in old *p16-INK-ATTAC* mice, in which systemic elimination of *p16^Ink4a+^* SnCs prevented age-related trabecular and cortical bone loss at the spine and femur by not only increasing bone formation but also by reducing bone resorption and suppressing bone marrow adiposity (27).

As anticipated, clearance of Sn osteocytes in old mice resulted in substantially reduced TAF^+^ osteocytes; however, it did not alter the percentage of total osteocyte empty lacunae and only modestly increased osteocyte apoptosis. Furthermore, specific elimination of Sn osteocytes markedly improved the quality of the osteocyte LCN as reflected by the number of osteocyte dendrites and their density. Although these findings may at first seem paradoxical, based on our previous work (26), we know that only a small percentage of osteocytes (~10% or less) become Sn with aging, even when studying mice late in life (e.g., at 24 months). Therefore, because AP treatment in *INK-ATTAC* mice does not clear all Sn osteocytes (i.e., only ~30% reduction), this ultimately results in a very limited proportion of osteocytes being eliminated among the overall vast network. Nevertheless, despite only clearing this very small population of dysfunctional osteocytes following 4 months of treatment, there were clear benefits to trabecular bone at the spine, and our short-term Sn osteocyte clearance study showed a profound local SASP reduction (using bone mRNA analyses) following just 2 weeks, which is likely the key driver of the beneficial skeletal changes observed and may also produce a more permissive environment for osteocytes to refill empty lacunae. Moreover, we hypothesize that suppressing the SASP in the bone milieu has additional benefits, beyond the scope of our study, for improving skeletal health in aging. For example, Sn osteocyte clearance significantly improved the quality of the osteocyte LCN, and given the recent demonstration that the LCN architecture is a primary determinant of the healthy skeletal response to mechanical loads (45), our data suggest that therapeutic strategies to eliminate SnCs and suppress the SASP could represent effective approaches to restore osteocyte function and health in old age.

Although the SASP was suppressed in whole bones with both local (*DMP*) and systemic (*INK*) senolysis, a direct comparison between the 2 AP-treated groups (*DMP* vs. *INK*) demonstrated a broader downregulation of SASP- and Sn-related genes and pathways (e.g., senescence, autophagy, p53 signaling) with systemic SnC clearance. As might be expected, local senolysis in bone predominantly affected the Wnt and several related pathways. In addition, in response to SnC clearance in both models, we observed a reduction in *Sost*, indicating that an anti-sclerostin–mediated anabolic mechanism led to increased bone formation following both local (*DMP*) and systemic (*INK*) senolysis. By contrast, *Rankl* was only reduced in whole bones following systemic SnC clearance, which raises the possibility that other SnC types, in addition to Sn osteocytes, are important sources of RankL in old age, although given the systemic nature of the *p16-INK-ATTAC* model, we cannot exclude the possibility that systemic clearance of SnCs through effects on other cell types leads to reduced bone resorption. The latter is supported by experiments showing that the SASP produced by Sn preadipocytes enhances osteoclastogenesis by promoting the survival of osteoclast precursors, which can be blocked pharmacologically using a JAK1/-2 inhibitor, and by in vivo studies demonstrating that inhibiting the SASP in old mice prevents bone loss and reduces bone resorption (27). Nevertheless, our findings establish that Sn osteocytes are clearly not the only culprits mediating skeletal aging.

To definitively establish a systemic role for senescence, we transplanted nonskeletal SnCs (i.e., Sn fibroblasts) i.p. into young adult mice, which after 2 months resulted in hallmarks of skeletal aging, including bone loss, a reduction in osteoblasts, an increase in marrow adiposity, and induction of senescence in osteocytes within the host. While acknowledging that there is complex heterogeneity in the SASP among different SnC types (46), these cell transplantation studies, in combination with our in vitro mechanistic studies, establish a proof of concept that the SASP of nonskeletal SnCs can inhibit osteogenesis while simultaneously enhancing adipogenesis, providing evidence that the SASP has profound effects on MSC lineage allocation in bone. Moreover, while previous in vitro studies had shown that SnCs can propagate senescence to neighboring cells in a paracrine manner (47, 48), our study establishes that SnCs can induce senescence in vivo in cells in a distant organ (bone) using perhaps the most definitive assay for cellular senescence, i.e., TAF (24).

In summary, using both genetic and SnC transplantation models to either eliminate *p16^Ink4a+^* Sn cells in old age or induce an aging Sn milieu in bone in younger mice, our studies demonstrate that both Sn osteocytes and nonskeletal SnCs contribute to skeletal aging. In contrast to systemic SnC clearance (27), Sn osteocyte clearance in old mice only partially prevented age-related bone loss by improving bone formation through a mechanism that involves both suppression of the SASP and sclerostin. We also found that systemic, but not local osteocyte-specific, SnC clearance reduced RankL, which indicates that other cells, in addition to Sn osteocytes, are important mediators of bone resorption in aging. Further, we demonstrate that the SASP caused an alteration in lineage commitment of MSCs toward the osteoblast and away from the adipocyte lineage, which was reversed with systemic SnC clearance, but not with Sn osteocyte–specific clearance. We complemented these genetic approaches with transplantation of Sn cells into the peritoneal cavity of young mice and demonstrated that not only did this lead to systemic bone loss through the SASP of these cells but, remarkably, actually induced osteocyte senescence within bone, which was assessed using the most rigorous assay currently available for defining Sn cells: the presence of TAFs. To our knowledge, this is the first evidence of Sn cells inducing “senescence at a distance” using these rigorous criteria. Finally, we establish the utility of *p16-LOX-ATTAC* mice, which will be useful in future studies when crossed with other mouse *Cre* drivers for dissecting the contributions of additional specific SnC types in mediating age-related tissue dysfunction. Thus, the evidence presented here best fits a model whereby SnCs promote aging through both cell-autonomous and -nonautonomous effects, therefore establishing that the most effective senolytics (or combinations thereof) will systemically eliminate SnCs to extend healthy aging.

## Methods

### General experimental approaches.

Mice were randomly assigned to experimental groups, as specified below. Equal numbers of mice representing each sex were balanced between treatment groups. No mice or samples were excluded from the analyses, which were conducted in a blinded fashion.

### Mice.

As detailed here and in the figure legends, various mouse models (both females and males), all on the *C57BL/6* background, were used in experiments including the following: *C57BL/6* WT, *p16-INK-ATTAC* (9, 10), and *p16-LOX-ATTAC* mice (see below for details). All mice, including *Cre* recombinase (*Cre*) mice, were maintained on the *C57BL/6* background. The *p16-LOX-ATTAC* mice were either noncrossed (controls) or crossed with *DMP1-Cre* (for mature osteoblasts and osteocytes) (14) mice as well as *CMV-Cre* (ubiquitous expression) (30), *E2a-Cre* (ubiquitous expression) (31), or *β-actin–Cre* (ubiquitous expression) (36) mice, which targets the expression of *Cre* to the early mouse embryo, thus causing *Cre*-mediated recombination in various tissues. Mice were housed in ventilated cages within an accredited facility under a 12-hour light/12-hour dark cycle with a constant temperature (23°C) and had ad libitum access to water and food (standard mouse chow, Lab Diet 5053).

### p16-LOX-ATTAC mouse model.

In order to achieve cell-specific expression of *ATTAC* and thereby enable the conditional elimination of specific SnC types, David G. Monroe (Mayo Clinic, Rochester, Minnesota, USA) developed a construct in which the same *p16^Ink4a^* promoter (devised by Wang et al., ref. 49) used in the *p16-INK-ATTAC* model (9, 10) drives expression of a lox-stop-lox (LSL) cassette that includes EGFP and three SV40 poly(A) transcription termination sequences (3xSTOP) flanked by *loxP* sites, followed by the *ATTAC* cassette (devised by P. Scherer, ref. 29), which encodes a FLAG-tagged FKBP-Casp8 fusion protein. This construct was made by first digesting the attB-containing pBT378 plasmid (50) with HindIII and NotI and cloning in a linker containing additional SbfI and NsiI sites for subsequent cloning steps. The mouse *p16^Ink4a^* promoter, encompassing –2617 to +81 relative to the transcription start site, was amplified using LongAmp Taq DNA polymerase (New England BioLabs) and cloned into the HindIII site. Next, the LSL cassette was produced as a gBLOCK (Integrated DNA Technologies [IDT]) and cloned into the 3′ HindIII and SbfI sites. Finally, the *ATTAC* cassette, followed by a bGH-polyA signal, was produced as a gBLOCK and cloned into the SbfI and NsiI sites. Transgenic mice were produced through the Stanford Transgenic, Knockout and Tumor Model Center by selectively inserting the *p16-LOX-ATTAC* construct into the site-specific Rosa26 locus in C57BL/6 mice using integrase-mediated transgenesis (50). This technique ensures high-efficiency, single-copy transgene insertion into a predetermined and transcriptionally active chromosomal locus. In the unrecombined state (no *Cre*), transgene-harboring mice express *EGFP* but not the FLAG-tagged FKBP-caspase 8 (Casp8) fusion protein; however, when crossed with a *Cre* model, the *EGFP* and 3xSTOP cassette are removed, resulting in expression of the FLAG-tagged FKBP-Casp8 fusion protein. Thus, in cells with an active *Cre* recombinase, there is no *EGFP* expression, but the FLAG-tagged FKBP-Casp8 transgene is expressed. Administration of AP to *LOX-ATTAC* mice crossed with a *Cre* model prompts activation of the FKBP/Casp8 apoptosis cascade in a specific SnC type (*Cre*-dependent) to allow for the cells’ inducible “suicide.” Importantly, the combination of *Cre* and AP permitted us to attain both cell-specific and temporal (in old mice after the onset of age-related bone loss) control of SnC elimination.

### DoxR-induced senescence in vivo study.

This study is described in detail in the Supplemental Methods.

### Mouse harvests and tissue collections.

Mice were harvested and tissues were collected as previously described by our group (27) and as detailed in the Supplemental Methods.

### Biochemical assays.

All biochemical assays were performed in a blinded fashion, as detailed in the Supplemental Methods.

### Multiplex protein analyses.

Multiplexing analysis was performed using the Luminex 100 system (Luminex) by Eve Technologies, as detailed in the Supplemental Methods.

### Body composition assessments.

All body composition assessments were performed on mice that were nonanesthetized and conscious, as detailed in the Supplemental Methods.

### Skeletal assessments. Skeletal imaging.

All imaging and analysis were performed in a blinded fashion as described by our group previously (27) and as detailed in the Supplemental Methods.

### Skeletal histomorphometric assessments.

All bone histomorphometric analyses were performed in a blinded fashion as previously described by our group (27) and as detailed in the Supplemental Methods.

### TUNEL assay to detect apoptotic osteocytes.

These methods are described in detail in the Supplemental Methods.

### Osteocyte LCN analysis.

These methods are described in detail in the Supplemental Methods.

### CM generation.

The generation of CM was described previously by our group (27) and is detailed in the Supplemental Methods.

### In vitro osteoblast and adipocyte differentiation.

These methods are detailed in the Supplemental Methods.

### Immunostaining for FLAG.

These methods are detailed in the Supplemental Methods.

### RT-qPCR analyses.

Details on the RT-qPCR procedures and analyses are provided in the Supplemental Methods.

### TAF.

To measure osteocyte or osteoblast cellular senescence, as described previously (51), the TAF assay was performed on nondecalcified, methacrylate-embedded bone sections, as detailed in the Supplemental Methods.

### Data availability.

All data are provided in the supplemental materials as source data or supplemental data.

### Statistics.

Graphical data are shown in dot plots as the mean ± SEM unless otherwise specified. No mice or samples were excluded from the statistical analyses. Data were examined for distribution and normality (skewness and kurtosis) using histograms and dot plots. When the normality or equal variance assumptions for parametric analysis methods were not met, data were analyzed using nonparametric statistics (e.g., Wilcoxon rank-sum test). For parametric tests, as dependent on the comparison, between-groups differences were analyzed using either an independent samples Student’s *t* test or 1-way ANOVA, as statistically justified and specified in the figure legends. If a statistically significant (*P* < 0.05) effect was determined by ANOVA, pairwise multiple comparisons were corrected as appropriate using the Tukey post hoc method. Comparison of prespecified groups of gene sets or cytokine array data was performed using a MANOVA model as previously validated for such comparisons (52). Group sample sizes were based on previously published experiments in our laboratory (27, 51), where statistically significant (*P* < 0.05) differences were observed for various senescence- and skeletal-related endpoints in response to multiple interventions. For heatmaps, the *z* scores were calculated using the ΔCt values with a transformation according to the following formula: *Z* = (*X* – μ)/σ, where *Z* = standard score, *X* = observed value, μ = mean of the sample, and σ = standard deviation.

To detect significant differences among the 117 SenMayo genes, we chose a Student’s *t* test (2-tailed), with a cutoff of *P* < 0.05 (2-tailed) considered statistically significant. The BioVenn diagram was designed as recommended by Hulsen et al. (53), whereas pathway analysis was performed using gene ontology, while choosing the BioPlanet 2019 pathways (Enrichr, BioPlanet 2019; ref. 54) and focusing on pathways with a Benjamini-Hochberg correction for multiple testing of adjusted *P* < 0.05 (*P*_adj_ < 0.05). The combined score was calculated by taking the log of each *P* value and multiplying by the *z* score from the expected rank. Group sizes and sex (female, male, or both) are specified for each experiment in the corresponding figure Legends. To eliminate sex as a potential confounder, in analyses in which females and males were combined, equal (or as close as possible) numbers of mice representing each sex were balanced between the treatment groups. In addition, for the primary endpoints (μCT outcomes in the different mouse models), the key data are shown separately for female and male mice (including the main and supplemental figures). As recommended by Garcia-Sifuentes and Maney (35), we also used the ANOVA model testing for a sex × treatment interaction, and if this was not significant (*P* > 0.05, as was the case for all AVOVAs, as shown in Supplemental Table 1), the pooled data are presented. For the secondary analyses, following a test for a sex × treatment interaction, the pooled data are presented if the interaction was not significant (*P* > 0.05). Statistical analyses were performed using either GraphPad Prism, version 9.3 (GraphPad Software) or the Statistical Package for the Social Sciences for Windows, version 25.0 (SPSS), with *P* < 0.05 (2-tailed) considered statistically significant.

### Study approval.

Animal studies were performed under IACUC-approved protocols, and experiments were executed in accordance with Mayo Clinic IACUC guidelines and regulations.

## Author contributions

JNF, DGM, and SK conceived the studies and designed experiments. JNF and SK directed and supervised all aspects of the study and take responsibility for the integrity of the data analysis. JNF, DS, MLD, JK, JLR, SJV, MNF, ABL, and YZ performed experiments. JNF and DS analyzed data. JNF and SK interpreted the data. DGM designed the *p16-LOX-ATTAC* transgene construct. JNF wrote the initial manuscript, and JNF, DS, MLD, JK, JLR, SJV, MNF, ABL, YZ, MW, YI, RJP, LJN, PDR, DJ, JFP, NKL, TT, JLK, DGM, and SK contributed ideas and participated in editing and revising the manuscript.

## Supplementary Material

Supplemental data

Supplemental supporting data values

## Version 1 02/21/2023

In-Press Preview

## Version 2 04/17/2023

Electronic publication
