# Supplementary material for: Local senolysis in aged mice only partially replicates the benefits of systemic senolysis
Source: J Clin Invest. 2023 Apr 17;133(8):e162519. doi: 10.1172/JCI162519 (PMC10104901; doi:10.1172/JCI162519)
Supplement: Supplemental data [file jci-133-162519-s021.pdf]

Supplemental Figure Legends

Supplemental Figure 1. Changes in murine *p16Ink4a* mRNA expression and the SASP in osteocytes with aging. (**A**) Changes in murine mRNA expression of the chronic senescence effector, $p16^{Ink4a}$, throughout the lifespan (at 1-mo [n=5♀, n=5♂], 6-mo [n=14♀, n=12♂], 12-mo [n=5♀, n=5♂], 18-mo [n=14♀, n=10♂], and 24-mo [n=9♀, n=10♂] of age) by RT-qPCR analysis in OCY-enriched bone samples obtained from *C57BL/6 Wild-Type* (*WT*) mice; comparisons are relative to the young adult (6-mo) mice (ANOVA, followed by Tukey *post hoc* test to adjust for multiple comparisons), which is the most appropriate young reference group. (**B**) *In vivo* changes in mRNA expression of the SASP in osteocyte-enriched bone preparations between Young (6-mo, white bars; n=12) adult versus Old (24-mo, black bars; n=10) *WT C57BL/6* mice. Data represent Mean ± SEM (error bars). *P < 0.05; **P < 0.01; ***P < 0.001 (independent samples t-test or Wilcoxon rank-sum test, as appropriate). Abbreviations: SASP = senescence-associated secretory phenotype (SASP). Lifespan timecourse data are reproduced from (Farr et al. *Nat Med* 23(9):1072-9, 2017). Young versus Old data are reproduced from (Farr et al. *J Bone Miner Res* 31(11):1920-9, 2016).

Supplemental Figure 2. Generation and validation of *p16-LOX-ATTAC* mice. (**A**) Schematic of the *p16-LOX-ATTAC* construct. (**B**) Cross between *p16-LOX-ATTAC* (un-recombined) and *CMV-Cre+/-* (ubiquitous expression) mice. (**C**) RT-qPCR mRNA expression of *EGFP* in BMSCs obtained from *p16-LOX-ATTAC* (un-recombined) or *CMV-Cre+/-;p16-LOX-ATTAC* mice; Note: *EGFP* is not detected (Not Expressed [NE]; all cycle threshold (Ct) values = 40) in BMSCs from *CMV-Cre+/-;p16-LOX-ATTAC* mice, following *Cre*-recombination. (**D**) IHC staining for FLAG in BMSCs isolated from *p16-LOX-ATTAC* (un-recombined) or *CMV-Cre+/-;p16-LOX-ATTAC* (recombined) mice; scale bar = 20μm. Data represent Mean ± SEM (error bars). *p < 0.05; **p < 0.01; ***p < 0.001 (independent samples t-test or Wilcoxon rank-sum test, as appropriate). Abbreviations: BMSCs = bone marrow stromal cells; IHC = immunohistochemistry. (**E**) Experimental design of the *in vivo p16-LOX-ATTAC* validation study, in which *E2a-Cre+/-;p16-LOX-ATTAC* mice, treated with Vehicle (Veh) or doxorubicin (DoxR) at days 0 and 10, were additionally randomized to Veh or AP treatments (10 mg/kg; twice weekly) for 24 days. (**F**) RT-qPCR mRNA expression of $p16^{Ink4a}$ in liver of female (♀) and male (♂) *E2a-Cre+/-;p16-LOX-ATTAC* mice (n=6-7 per group) following treatments at day 24. Data represent Mean ± SEM (error bars). $^{\dagger}p$ < 0.05 (ANOVA vs Veh followed by Tukey *post hoc* test); $^{\ddagger}p$ < 0.001 (ANOVA vs DoxR + Veh followed by Tukey *post hoc* test). Abbreviations: Veh = Vehicle; DoxR = Doxorubicin.

Supplemental Figure 3. Senescent (Sn) osteocytes (OCYs) are cleared by AP treatment in old female and male *DMP1-Cre+/-;p16-LOX-ATTAC* mice. (**A**) Study design for clearance of Sn OCYs in female (♀) and male (♂) old (20-mo) *DMP1-Cre+/-;p16-LOX-ATTAC* mouse cohorts, randomized to Vehicle (Veh: ♀ = pink, ♂ = light blue) or AP20187 (AP: ♀ = Red, ♂ = Dark Blue) for 4-mo. (**B**) RT-qPCR analysis of *p16Ink4a* mRNA expression across the indicated tissues in old *DMP1-Cre+/-;p16-LOX-ATTAC* mice (Veh: ♀ = pink, ♂ = light blue) or AP (♀= Red, ♂ = Dark Blue) at 24-mo of age, following 4-mo of treatment. Data represent mean SEM ± (error bars). ns = not significant (P>0.05); *P<0.05; **P<0.01; ***P<0.001 (independent samples *t*-Test or Wilcoxon's rank-sum test, as appropriate).

Supplemental Figure 4. Effects of local senescent (Sn) osteocyte (OCY)-specific clearance on body composition in old mice. Study design for the local clearance of Sn OCYs in female (♀) and male (♂) old (20-mo) *DMP1-Cre+/-;p16-LOX-ATTAC* mouse cohorts, randomized to Vehicle (Veh: n=11; 6♀, 5♂, grey) or AP20187 (AP: n=11; 6♀, 5♂ teal) for 4-mo.

(**A-D**) Body mass at (**A**) baseline (20-mo), (**B**) monthly thereafter, (**C**) endpoint (24-mo), and (**D**) percentage (%) change from baseline to endpoint. (**E-F**) Echo-MRI-derived total body fat mass at (**E**) baseline (20-mo) and (**F**) endpoint (24-mo). (**G-H**) Echo-MRI-derived total body lean mass at (**G**) baseline (20-mo) and (**H**) endpoint (24-mo). ns = not significant (*P*>0.05); (independent samples *t*-Test or Wilcoxon's rank-sum test, as appropriate).

Supplemental Figure 5. Effects of local senescent (Sn) osteocyte (OCY)-specific clearance on body composition in old female mice. Study design for the local clearance of Sn OCYs in female (♀) old (20-mo) *DMP1-Cre+/-;p16-LOX-ATTAC* mice, randomized to Vehicle (Veh: n=6♀, pink) or AP20187 (AP: n=6♀, red) for 4-mo. (**A-D**) Body mass at (**A**) baseline (20-mo), (**B**) monthly thereafter, (**C**) endpoint (24-mo), and (**D**) percentage (%) change from baseline to endpoint. (**E-F**) Echo-MRI-derived total body fat mass at (**E**) baseline (20-mo) and (**F**) endpoint (24-mo). (**G-H**) Echo-MRI-derived total body lean mass at (**G**) baseline (20-mo) and (**H**) endpoint (24-mo). ns = not significant (*P*>0.05); (independent samples *t*-Test or Wilcoxon's rank-sum test, as appropriate).

Supplemental Figure 6. Effects of local senescent (Sn) osteocyte (OCY)-specific clearance on body composition in old male mice. Study design for the local clearance of Sn OCYs in male (♂) old (20-mo) *DMP1-Cre+/-;p16-LOX-ATTAC* mice, randomized to Vehicle (Veh: n=5♂, pink) or AP20187 (AP: n=5♂, red) for 4-mo. (**A-D**) Body mass at (**A**) baseline (20-mo), (**B**) monthly thereafter, (**C**) endpoint (24-mo), and (**D**) percentage (%) change from baseline to endpoint. (**E-F**) Echo-MRI-derived total body fat mass at (**E**) baseline (20-mo) and (**F**) endpoint (24-mo). (**G-H**) Echo-MRI-derived total body lean mass at (**G**) baseline (20-mo) and (**H**) endpoint (24-mo). ns = not significant (*P*>0.05); (independent samples *t*-Test or Wilcoxon's rank-sum test, as appropriate).

Supplemental Figure 7. Effects of AP20187 treatment on the skeleton of old female *p16-LOX-ATTAC* (non-crossed control) mice. (**A**) Study design in old female (20-mo) *p16-LOX-ATTAC* (non-crossed control) mice randomized to Vehicle (Veh, pink) or AP20187 (AP, red) for 4-mo. (**B**) DXA-derived areal bone mineral density (aBMD, g/cm^2) at baseline in females (20-mo ♀). (**C-F**) Quantification of study endpoint (24-mo) µCT-derived (**C**) bone volume fraction (BV/TV; %), (**D**) trabecular number (Tb.N), (**E**) trabecular thickness (Tb.Th), (**F**) trabecular separation (Tb.Sp) at the lumbar spine in female mice treated with Veh (♀) vs AP (♀). (**G-L**). Quantification of µCT-derived (**G**) BV/TV, (**H**) cortical volumetric bone mineral density (vBMD), (**I**) cortical thickness (Ct.Th), (**J**) endocortical circumference (EC), (**K**) periosteal circumference (PC), and (**L**) µFEA-derived failure load at the femur metaphysis in female mice. Data represent mean SEM ± (error bars). ns = not significant (*P*>0.05); (independent samples *t*-Test or Wilcoxon's rank-sum test, as appropriate).

Supplemental Figure 8. Effects of AP20187 treatment on the skeleton of old male *p16-LOX-ATTAC* (non-crossed control) mice. (**A**) Study design in old (20-mo) male *p16-LOX-ATTAC* (non-crossed control) mice randomized to Vehicle (Veh, light blue) or AP20187 (AP, dark blue) for 4-mo. (**B**) DXA-derived areal bone mineral density (aBMD, g/cm^2) at baseline in males (20-mo; ♂). (**C-F**) Quantification of study endpoint (24-mo) µCT-derived (**C**) bone volume fraction (BV/TV; %), (**D**) trabecular number (Tb.N), (**E**) trabecular thickness (Tb.Th), (**F**) trabecular separation (Tb.Sp) at the lumbar spine in mice treated with Veh (♂) vs AP (♂). (**G-L**) Quantification of µCT-derived (**G**) BV/TV, (**H**) cortical volumetric bone mineral density (vBMD), (**I**) cortical thickness (Ct.Th), (**J**) endocortical circumference (EC), (**K**) periosteal circumference (PC), and (**L**) µFEA-derived failure load at the femur metaphysis in males. Data represent mean SEM ± (error bars). ns = not significant (*P*>0.05); (independent samples *t*-Test or Wilcoxon's rank-sum test, as appropriate).

Supplemental Figure 9. Effects of AP20187 treatment on the skeleton of old *p16-LOX-ATTAC* (non-crossed control) mice. (**A**) Study design in old (20-mo) *p16-LOX-ATTAC* (non-crossed control) mice, females and males combined, randomized to Vehicle (Veh, grey) or AP20187 (AP, teal) for 4-mo. (**B**) DXA-derived areal bone mineral density (aBMD, g/cm^2) at baseline (20-mo; n=7/group, ♀,♂). (**C-F**) Quantification of study endpoint (24-mo) µCT-derived (**C**) bone volume fraction (BV/TV; %), (**D**) trabecular number (Tb.N), (**E**) trabecular thickness (Tb.Th), (**F**) trabecular separation (Tb.Sp) at the lumbar spine in mice treated with Veh (n=7♀,♂) vs AP (n=7♀,♂). (**G-L**) Quantification of µCT-derived (**G**) BV/TV, (**H**) cortical volumetric bone mineral density (vBMD), (**I**) cortical thickness (Ct.Th), (**J**) endocortical circumference (EC), (**K**) periosteal circumference (PC), and (**L**) µFEA-derived failure load at the femur metaphysis (n=7♀,♂/group). Data represent mean SEM ± (error bars). ns = not significant (*P*>0.05); (independent samples *t*-Test or Wilcoxon's rank-sum test, as appropriate).

Supplemental Figure 10. Senescent cells (SnCs) are cleared by AP treatment in old *β-Actin-Cre+/-;p16-LOX-ATTAC* mice. (**A**) Study design for systemic clearance of SnCs in old (20-mo) *p16-LOX-ATTAC* x *β-Actin-Cre* mouse cohorts, males and females combined, randomized to vehicle (Veh, grey) or AP20187 (AP, teal) for 4-mo. (**B**) RT-qPCR analysis of *p16Ink4a* mRNA expression across tissues in mice (females [♀] and males [♂] combined, n=16-20 per tissue) treated with Veh (grey) vs AP (teal). (**C**) Study design for systemic clearance of SnCs in old (20-mo) *p16-LOX-ATTAC* x *β-Actin-Cre* mouse cohorts, males and females separately, randomized to vehicle (Veh: female ♀, pink; male ♂, light blue) or AP20187 (AP: female ♀, red; male ♂, dark blue) for 4-mo. (**D**) RT-qPCR analysis of *p16Ink4a* mRNA expression across tissues in mice (females [♀] n=9-11 per tissue and males [♂] n=6-10 per tissue, separately) treated with Vehicle (Veh: female ♀, pink; male ♂, light blue) or AP20187 (AP: female ♀, red; male ♂, dark blue). Data represent mean SEM ± (error bars). ns = not significant (*P*>0.05); **P*<0.05; ***P*<0.01 (independent samples *t*-Test or Wilcoxon's rank-sum test, as appropriate).

Supplemental Figure 11. Effects of systemic senescent cell (SnC) clearance on body composition and bone parameters in old *β-Actin-Cre+/-;p16-LOX-ATTAC* male mice. Study design for the systemic clearance of SnCs in male (♂) old (20-mo) *β-Actin-Cre+/-;p16-LOX-ATTAC* mice, randomized to Vehicle (Veh: n=9♂, light blue) or AP20187 (AP: n=9♂, dark blue) for 4-mo. (**A-D**) Body mass at (**A**) baseline (20-mo), (**B**) monthly thereafter, (**C**) endpoint (24-mo), and (**D**) percentage (%) change from baseline to endpoint. (**E-G**) Echo-MRI-derived total body fat mass at (**E**) baseline (20-mo), (**F**) endpoint (24-mo), and (**G**) % change from baseline to endpoint. (**H-J**) Echo-MRI-derived total body lean mass at (**H**) baseline (20-mo), (**I**) endpoint (24-mo), and (**J**) % change from baseline to endpoint. (**K**) DXA-derived areal bone mineral density (aBMD, g/cm^2) at baseline (20-mo; n=9♂/group). (**L**) Quantification of study endpoint (24-mo) µCT-derived bone volume fraction (BV/TV; %) at the lumbar spine in mice treated with Veh (n=9♂) vs AP (n=9♂). (**M-O**) Quantification of µCT-derived (**M**) BV/TV, (**N**) cortical thickness (Ct.Th), and (**O**) µFEA-derived failure load at the femur metaphysis (n=9♂/group). Data represent mean SEM ± (error bars). ns = not significant (*P*>0.05); **P*<0.05 (independent samples *t*-Test or Wilcoxon's rank-sum test, as appropriate).

Supplemental Figure 12. Effects of systemic senescent cell (SnC) clearance on body composition and bone parameters in old *β-Actin-Cre+/-;p16-LOX-ATTAC* female mice. Study design for the systemic clearance of SnCs in female (♀) old (20-mo) *β-Actin-Cre+/-;p16-LOX-ATTAC* mice, randomized to Vehicle (Veh: n=11♀, pink) or AP20187 (AP: n=11♀, red) for 4-mo. (**A-D**) Body mass at (**A**) baseline (20-mo), (**B**) monthly thereafter, (**C**) endpoint (24-mo), and (**D**) percentage (%) change from baseline to endpoint. (**E-G**) Echo-MRI-derived total body

fat mass at (**E**) baseline (20-mo), (**F**) endpoint (24-mo), and (**G**) % change from baseline to endpoint. (**H-J**) Echo-MRI-derived total body lean mass at (**H**) baseline (20-mo), (**I**) endpoint (24-mo), and (**J**) % change from baseline to endpoint. (**K**) DXA-derived areal bone mineral density (aBMD, g/cm^2) at baseline (20-mo; n=11♀/group). (**L**) Quantification of study endpoint (24-mo) µCT-derived bone volume fraction (BV/TV; %) at the lumbar spine in mice treated with Veh (n=11♀) vs AP (n=11♀). (**M-O**) Quantification of µCT-derived (**M**) BV/TV, (**N**) cortical thickness (Ct.Th), and (**O**) µFEA-derived failure load at the femur metaphysis (n=11♀/group). Data represent mean SEM ± (error bars). ns = not significant (*P*>0.05); **P*<0.05; ***P*<0.01 (independent samples *t*-Test or Wilcoxon's rank-sum test, as appropriate).

Supplemental Figure 13. Effects of systemic senescent cell (SnC) clearance on body composition and bone parameters in old *β-Actin-Cre+/-;p16-LOX-ATTAC* mice. Study design for the systemic clearance of SnCs in female (♀) and male (♂) old (20-mo) *β-Actin-Cre+/-;p16-LOX-ATTAC* mouse cohorts, randomized to Vehicle (Veh: n=20; 11♀, 9♂, grey) or AP20187 (AP: n=20; 11♀, 9♂ teal) for 4-mo. (**A-D**) Body mass at (**A**) baseline (20-mo), (**B**) monthly thereafter, (**C**) endpoint (24-mo), and (**D**) percentage (%) change from baseline to endpoint. (**E-G**) Echo-MRI-derived total body fat mass at (**E**) baseline (20-mo), (**F**) endpoint (24-mo), and (**G**) % change from baseline to endpoint. (**H-J**) Echo-MRI-derived total body lean mass at (**H**) baseline (20-mo), (**I**) endpoint (24-mo), and (**J**) % change from baseline to endpoint. (**K**) DXA-derived areal bone mineral density (aBMD, g/cm^2) at baseline (20-mo; n=20/group, 11♀,9♂). (**L**) Quantification of study endpoint (24-mo) µCT-derived bone volume fraction (BV/TV; %) at the lumbar spine in mice treated with Veh (n=20, 11♀,9♂) vs AP (n=20, 11♀,9♂). (**M-O**) Quantification of µCT-derived (**M**) BV/TV, (**N**) cortical thickness (Ct.Th), and (**O**) µFEA-derived failure load at the femur metaphysis (n=20, 11♀,9♂/group). Data represent mean SEM ± (error bars). ns = not significant (*P*>0.05); **P*<0.05; ***P*<0.01 (independent samples *t*-Test or Wilcoxon's rank-sum test, as appropriate).

Supplemental Figure 14. Comparisons of changes in SenMayo genes in response to local osteocyte-specific (*DMP*) versus systemic (*INK*) senescent cell (SnC) clearance. SenMayo was utilized to examine earlier changes in the senescence-associated secretory phenotype (SASP) and Sn-related transcriptome (after only two weeks of AP20187 [AP]) in whole-bones of old mice following local Sn osteocyte-specific (*DMP1-Cre+/-;p16-LOX-ATTAC*) or systemic (*p16-INK-ATTAC*) SnC clearance. To detect significant differences among the 117 SenMayo genes between each AP-treated group and their respective Veh-treated group, we chose a student *t*-Test (two-tailed) with a cut-off of **P*<0.05 (two-tailed) considered statistically significant. Comparison of pre-specified groups of gene sets was performed using a multivariate analysis of variance (MANOVA) model with a cut-off of **P*<0.05 (two-tailed) considered statistically significant.

Supplemental Figure 15. Comparisons of changes in cytokine array protein targets (n=32) in response to local osteocyte-specific (*DMP*) versus systemic (*INK*) senescent cell (SnC) clearance. Comparison of pre-specified groups of protein targets was performed using a multivariate analysis of variance (MANOVA) model with a cut-off of **P*<0.05 (two-tailed) considered statistically significant.

Supplemental Figure 16. Comparisons of changes in cytokine array protein targets, *i.e.* only those included in SenMayo (*Nat Commun* 13:4827, 2022), in response to local osteocyte-specific (*DMP*) versus systemic (*INK*) senescent cell (SnC) clearance. Comparison of pre-specified groups of protein targets was performed using a multivariate analysis of variance (MANOVA) model with a cut-off of **P*<0.05 (two-tailed) considered statistically significant.

Supplemental Figure 17. Osteocyte (OCY) lacunocanicular network (LCN) analysis and scoring system. Semiqualitative analysis of mouse OCY LCN using a 1-5 scaling system. Confocal OCY LCN images of the femur were scored using a semiquantitative 1-5 scale, where one represented a poorly connected OCY LCN whereas five represented a high quality OCY LCN, as depicted in the Figure. Image (**1**) Old mouse bone displaying poor canicular connectivity among OCYs; Images (**2-4**) Increasing progression of better OCY LCN connectivity; Image (**5**) Mouse bone with viable, robust ‘young-like’ OCY LCN connectivity. All analyses were performed in a blinded fashion.

Supplemental Figure 1

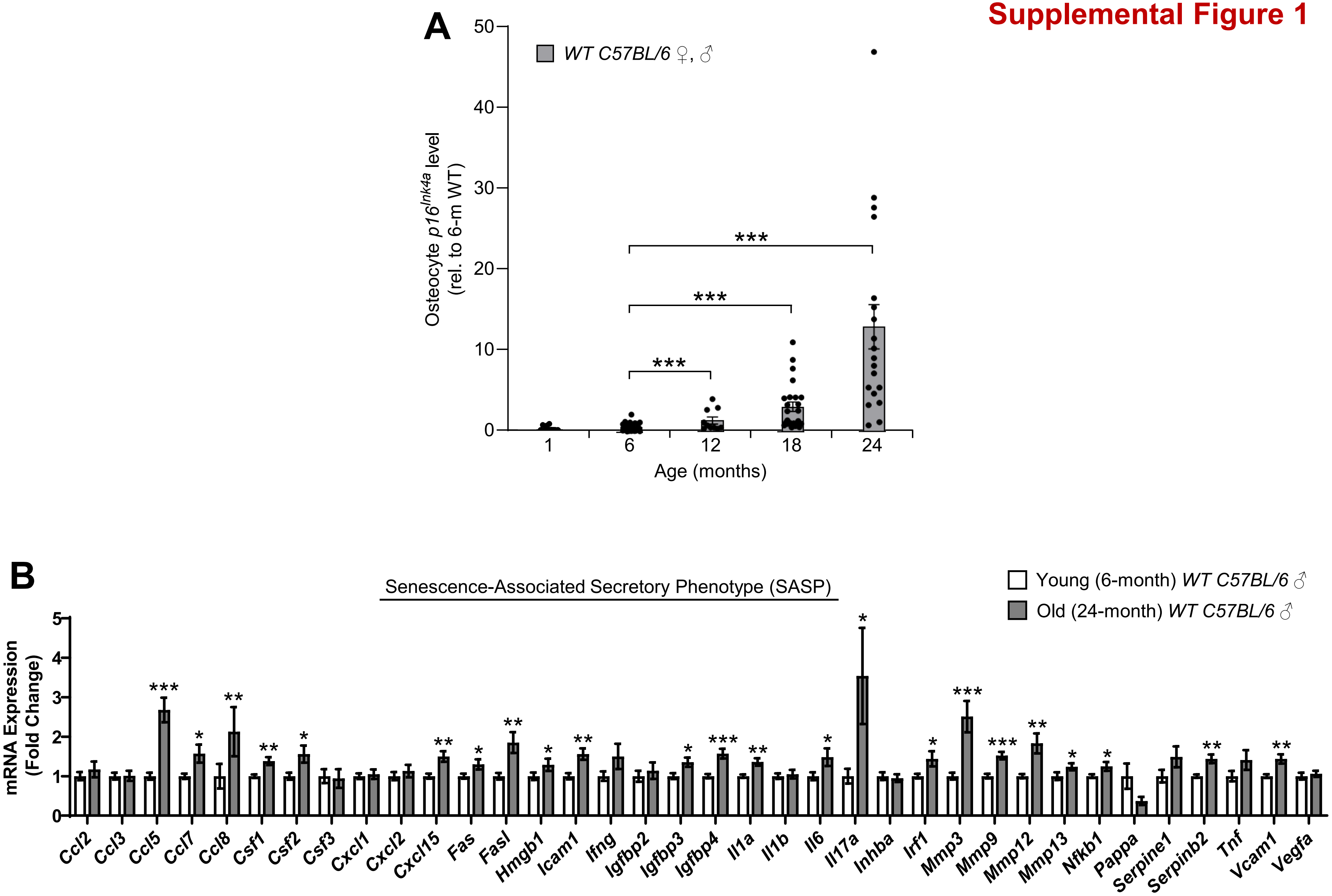

Supplemental Figure 2

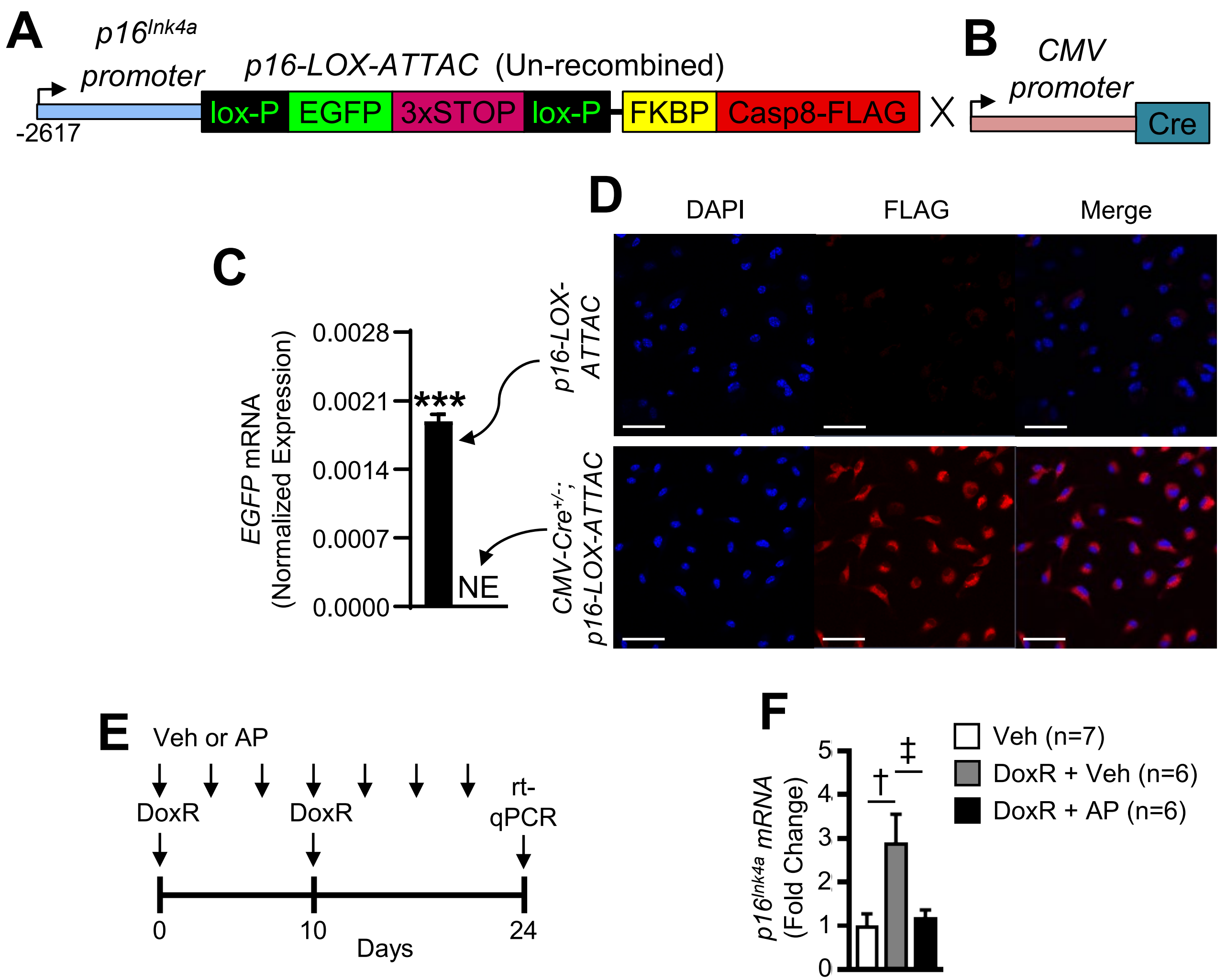

Supplemental Figure 3

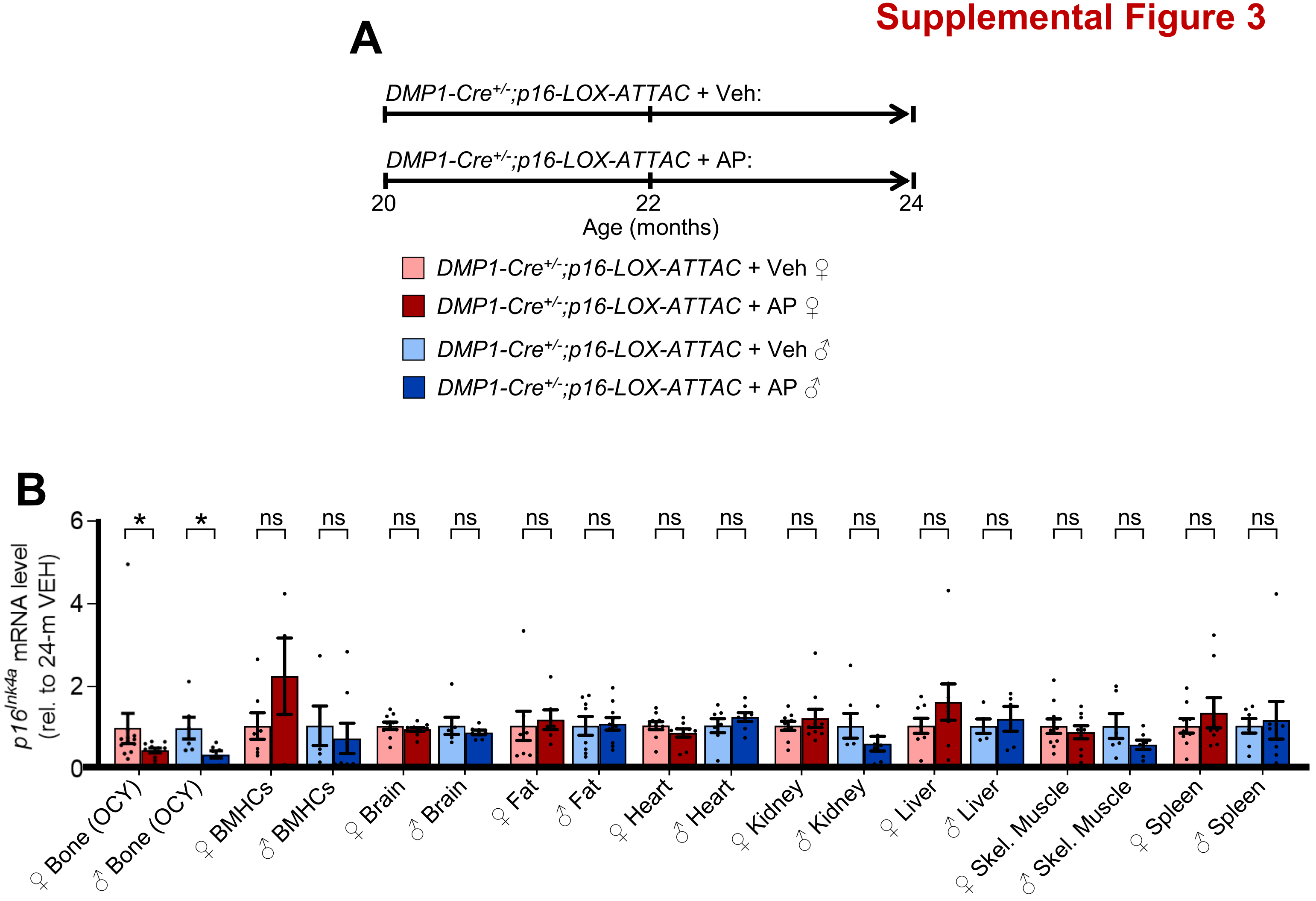

Supplemental Figure 4

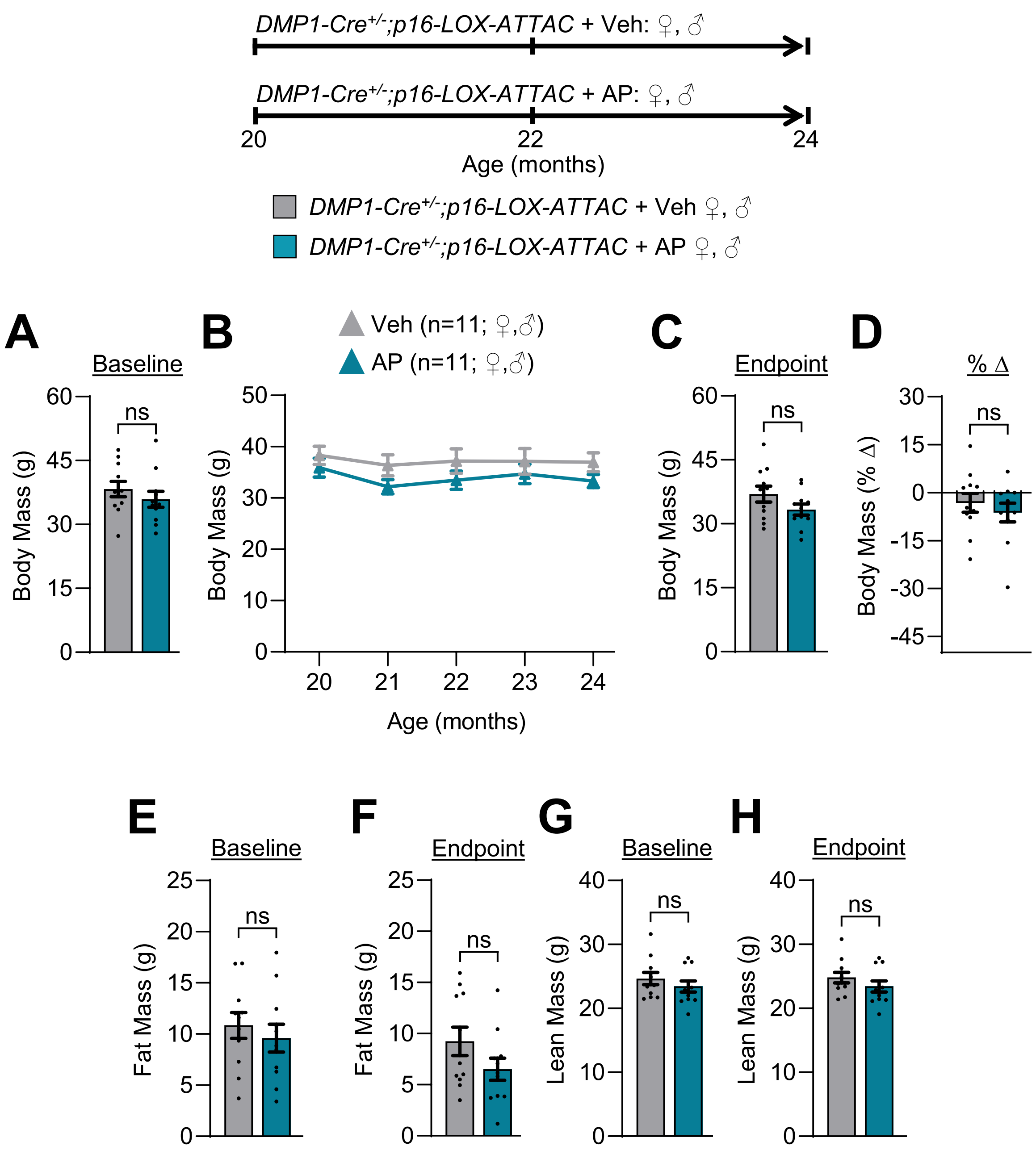

Supplemental Figure 5

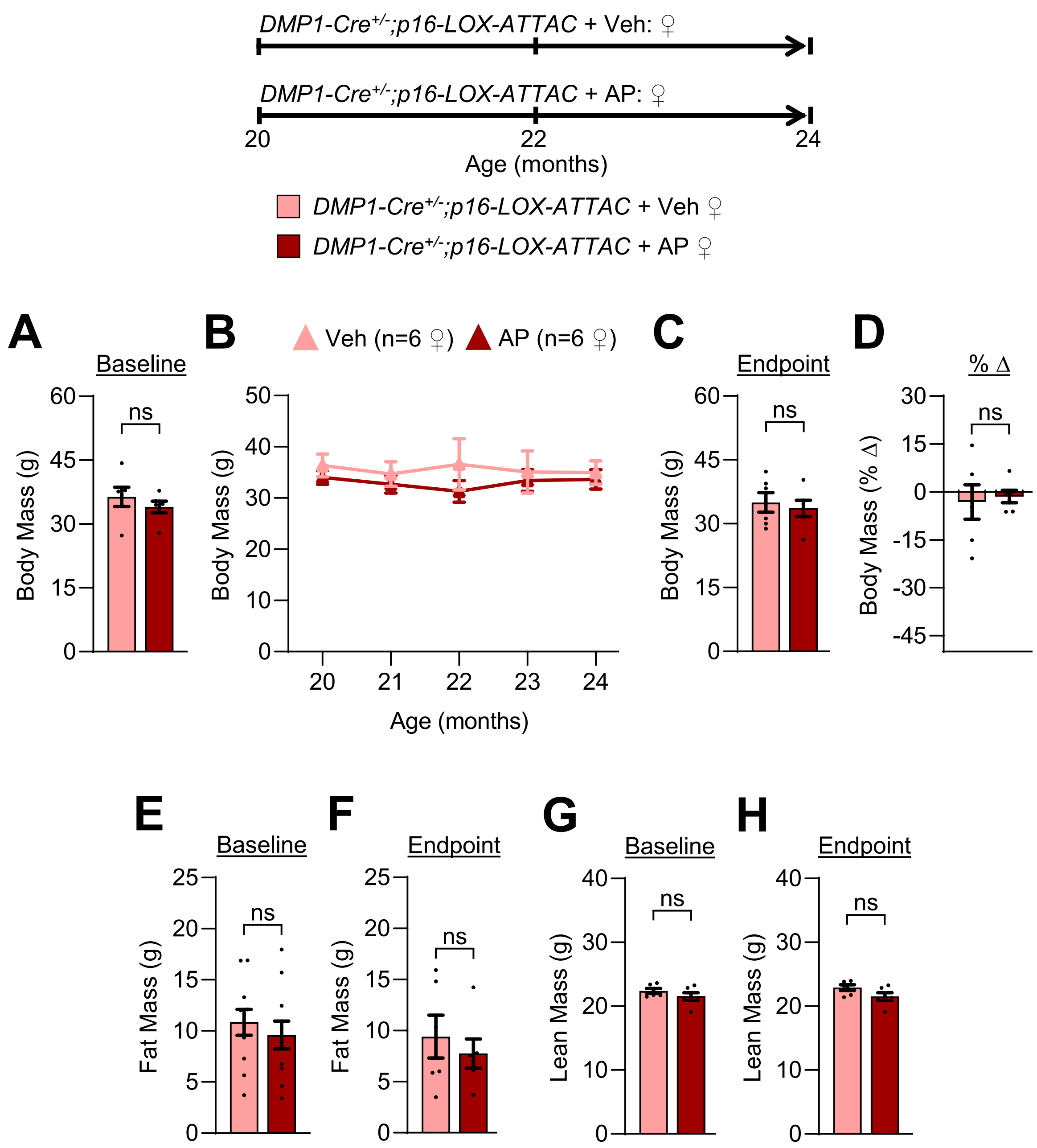

Supplemental Figure 6

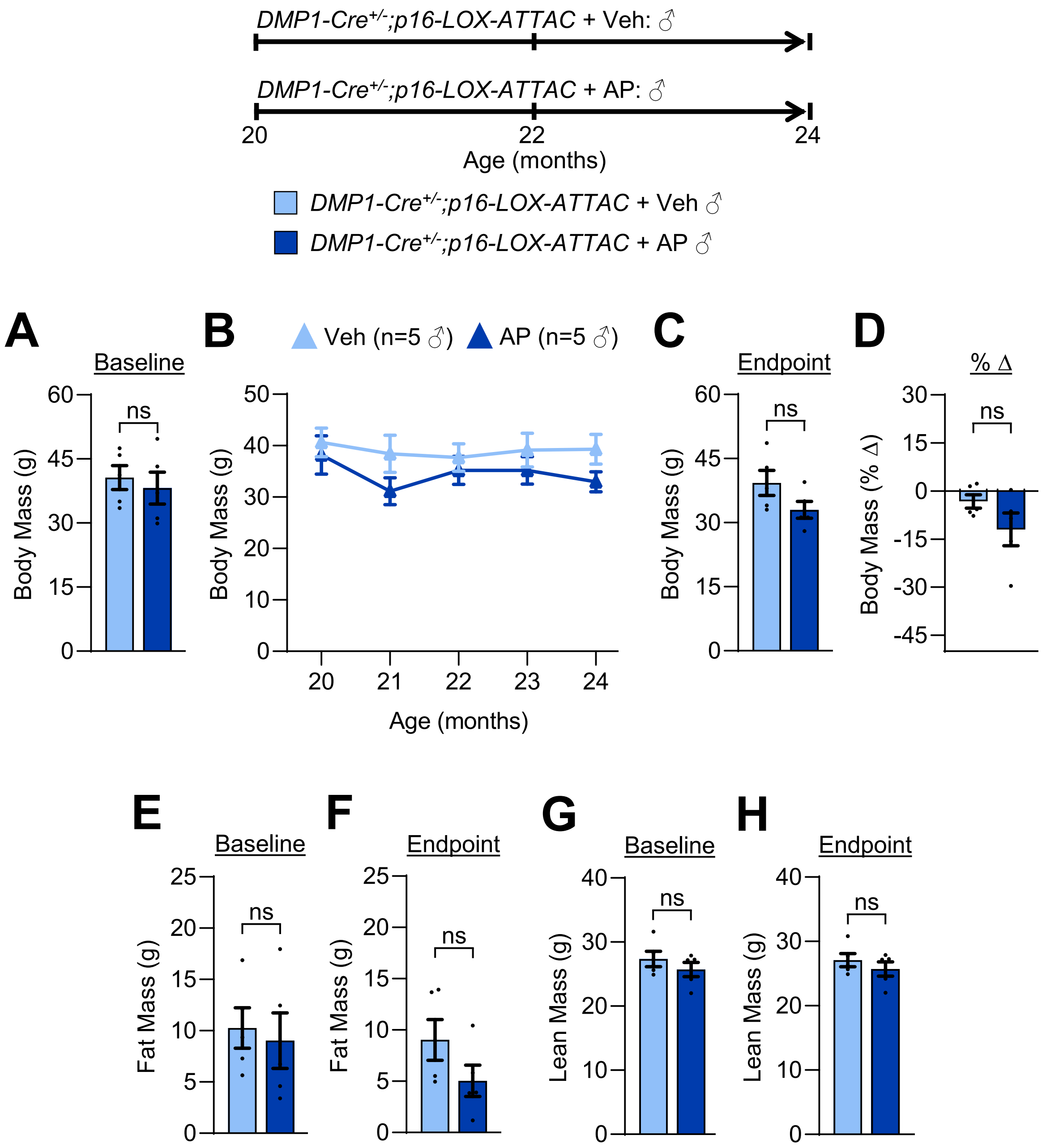

Supplemental Figure 7

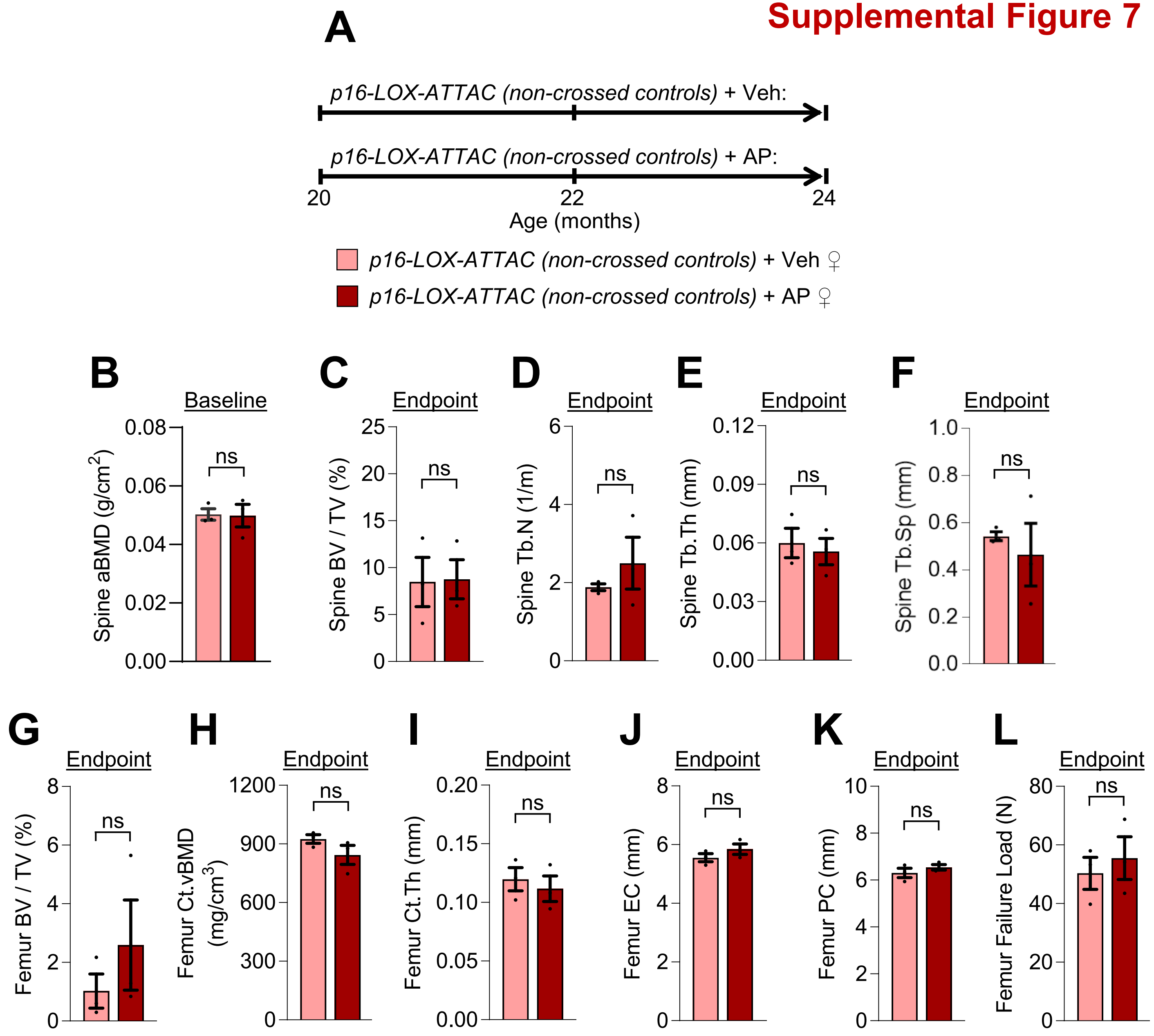

Supplemental Figure 8

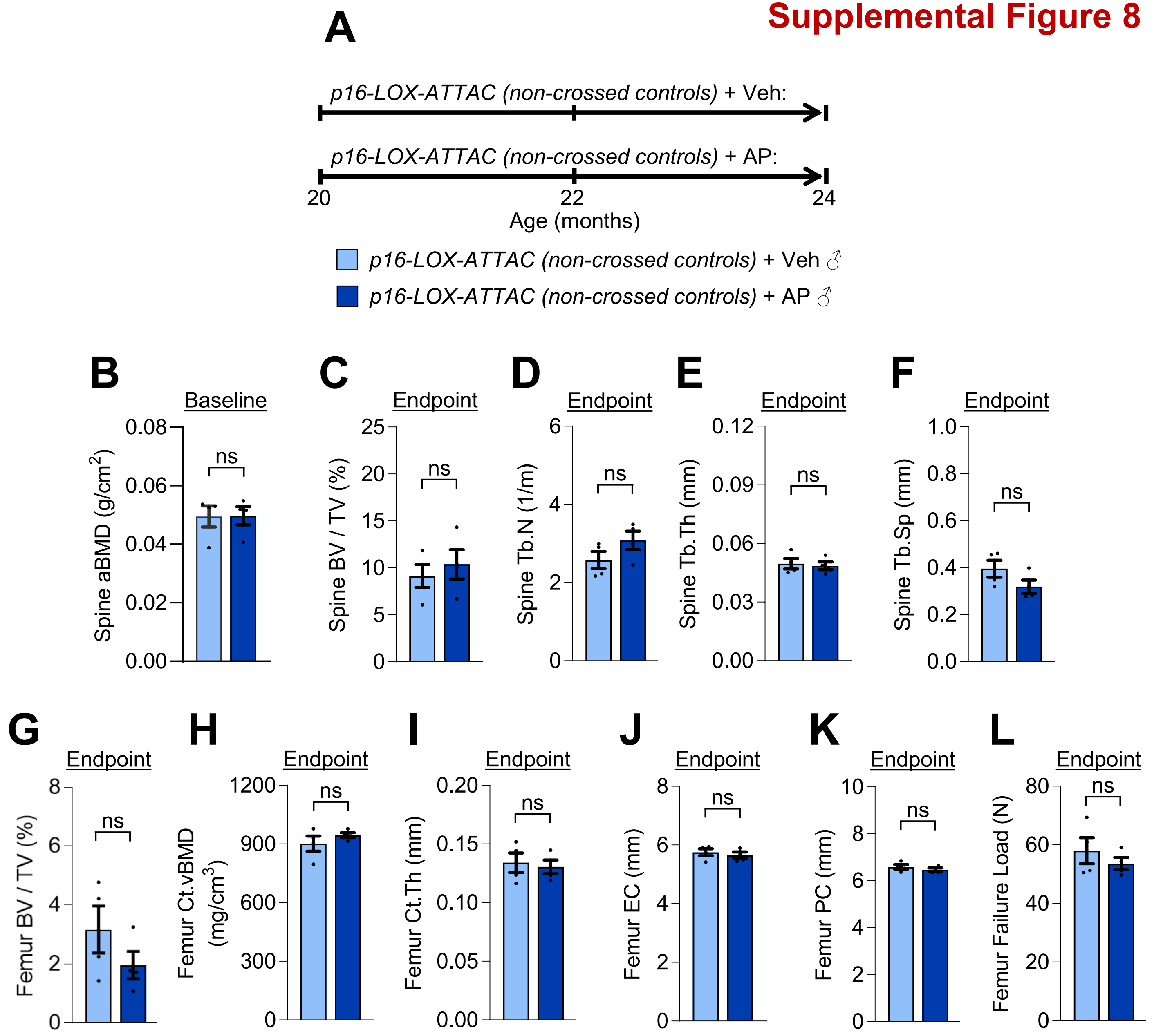

Supplemental Figure 9

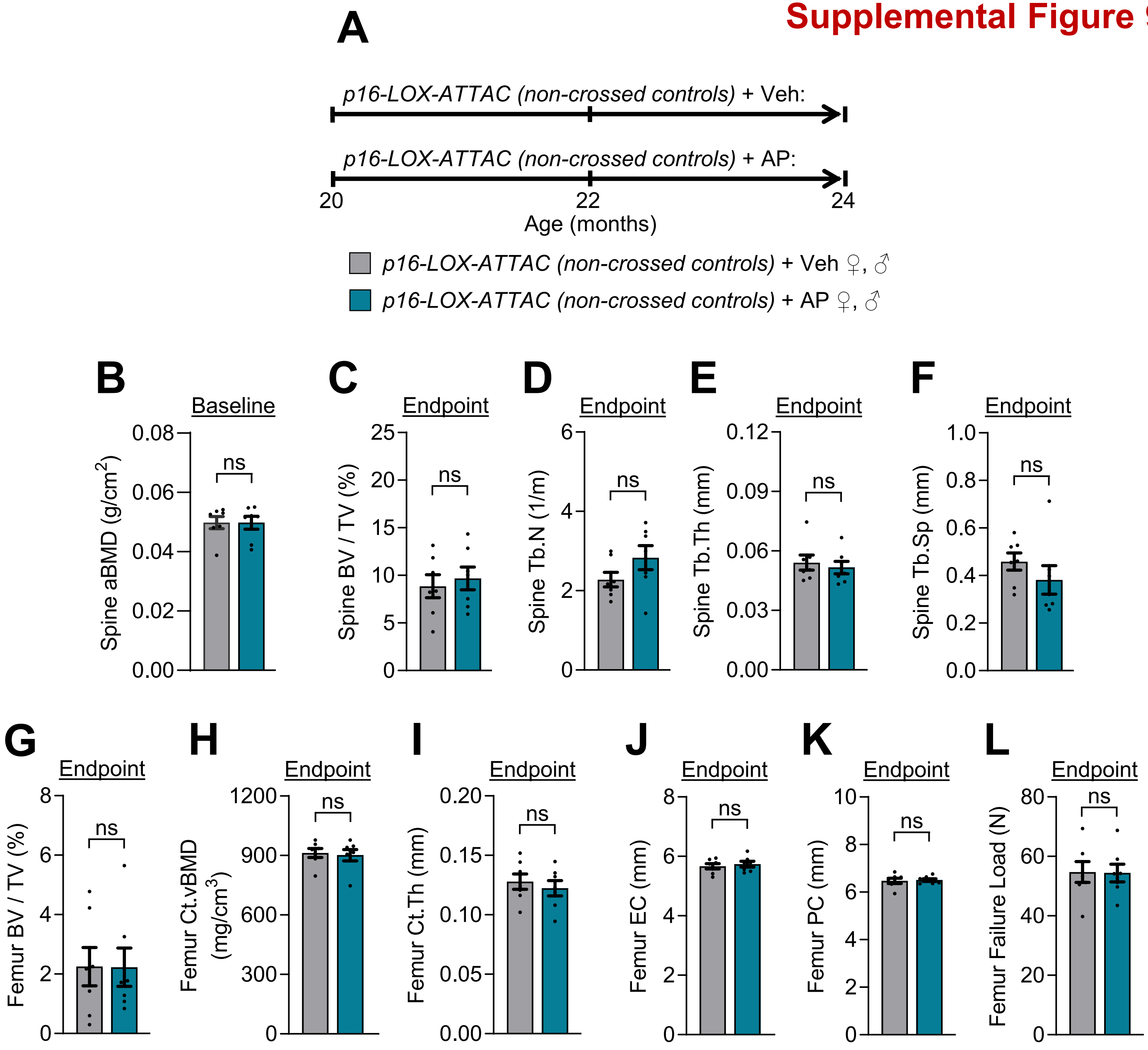

Supplemental Figure 10

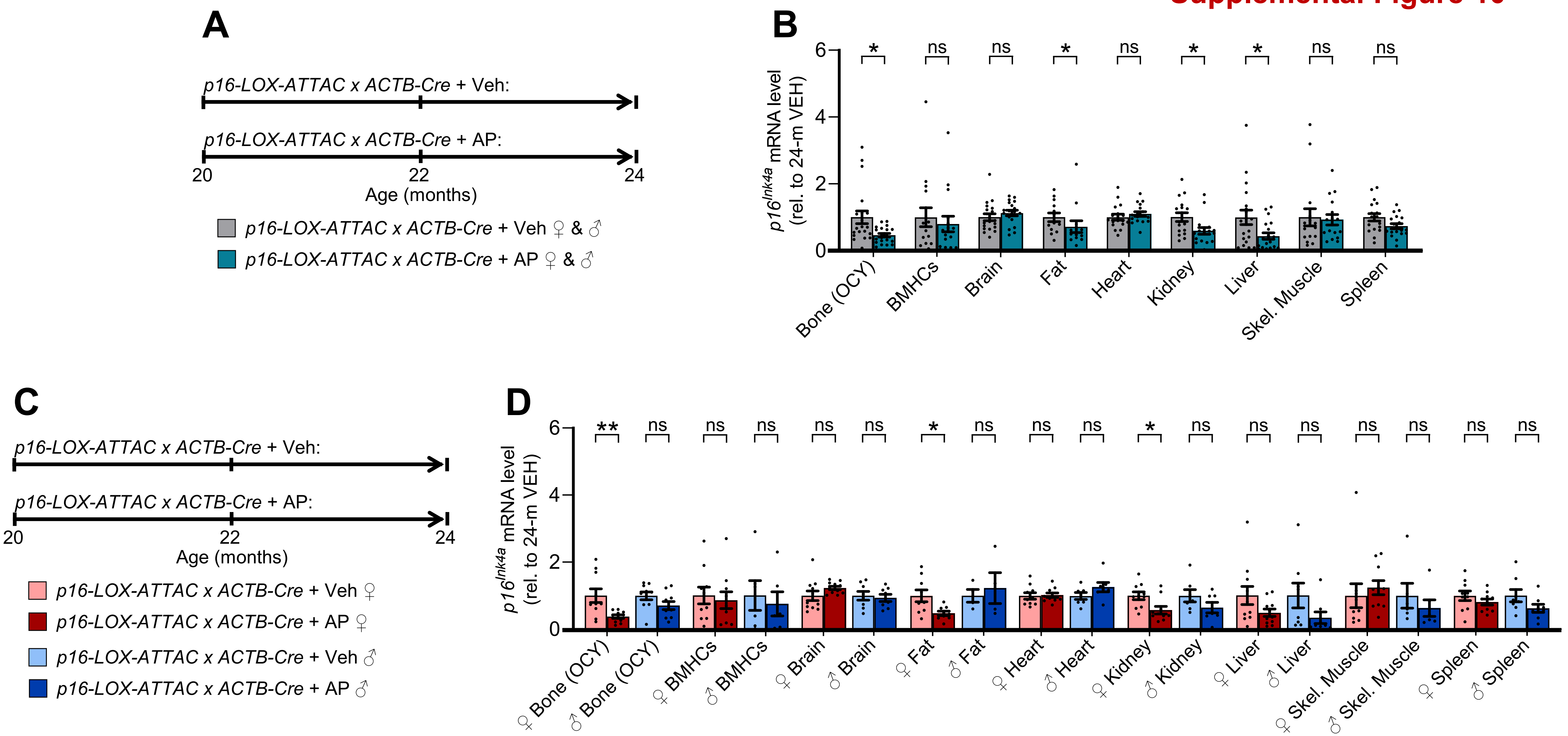

Supplemental Figure 11

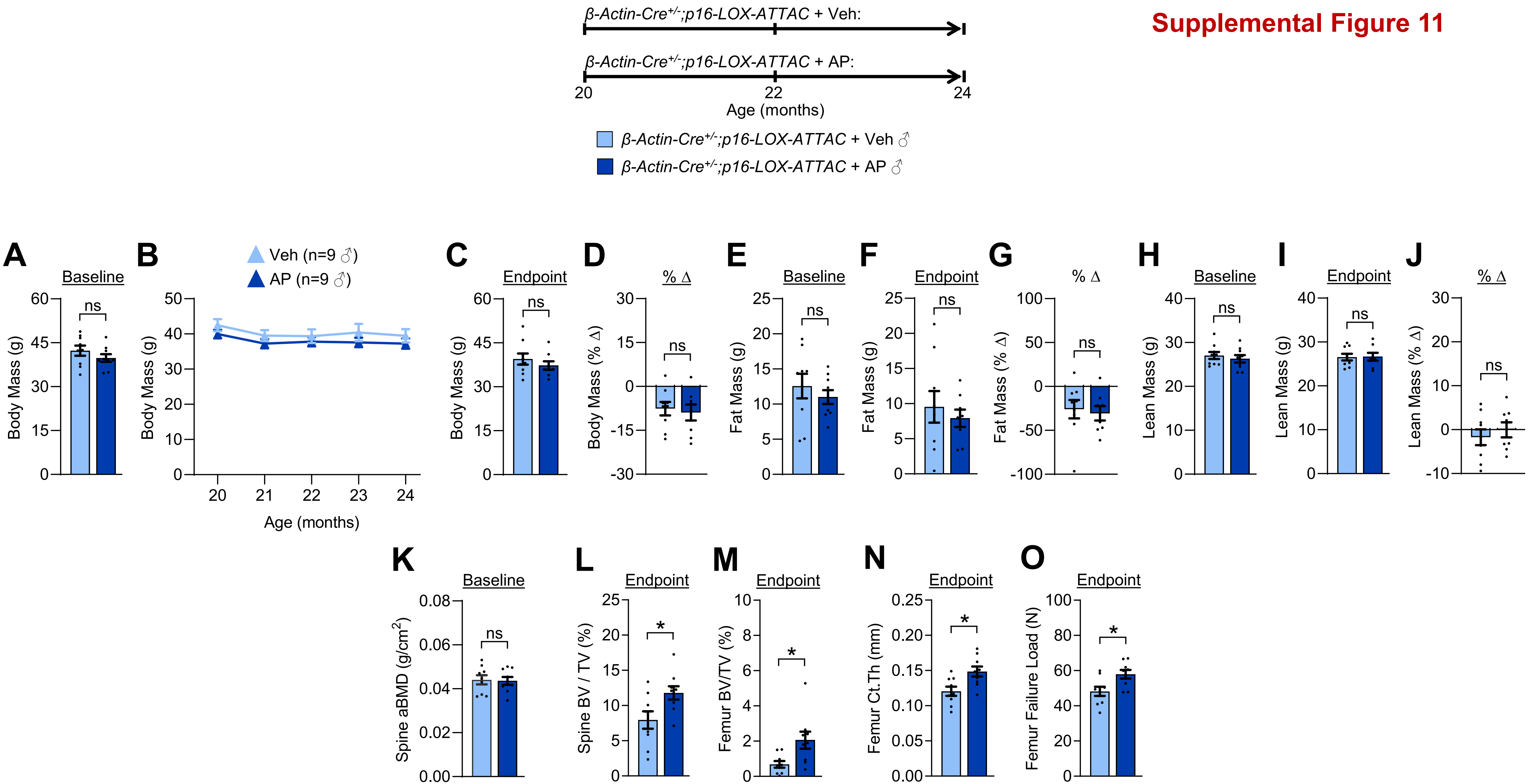

Supplemental Figure 12

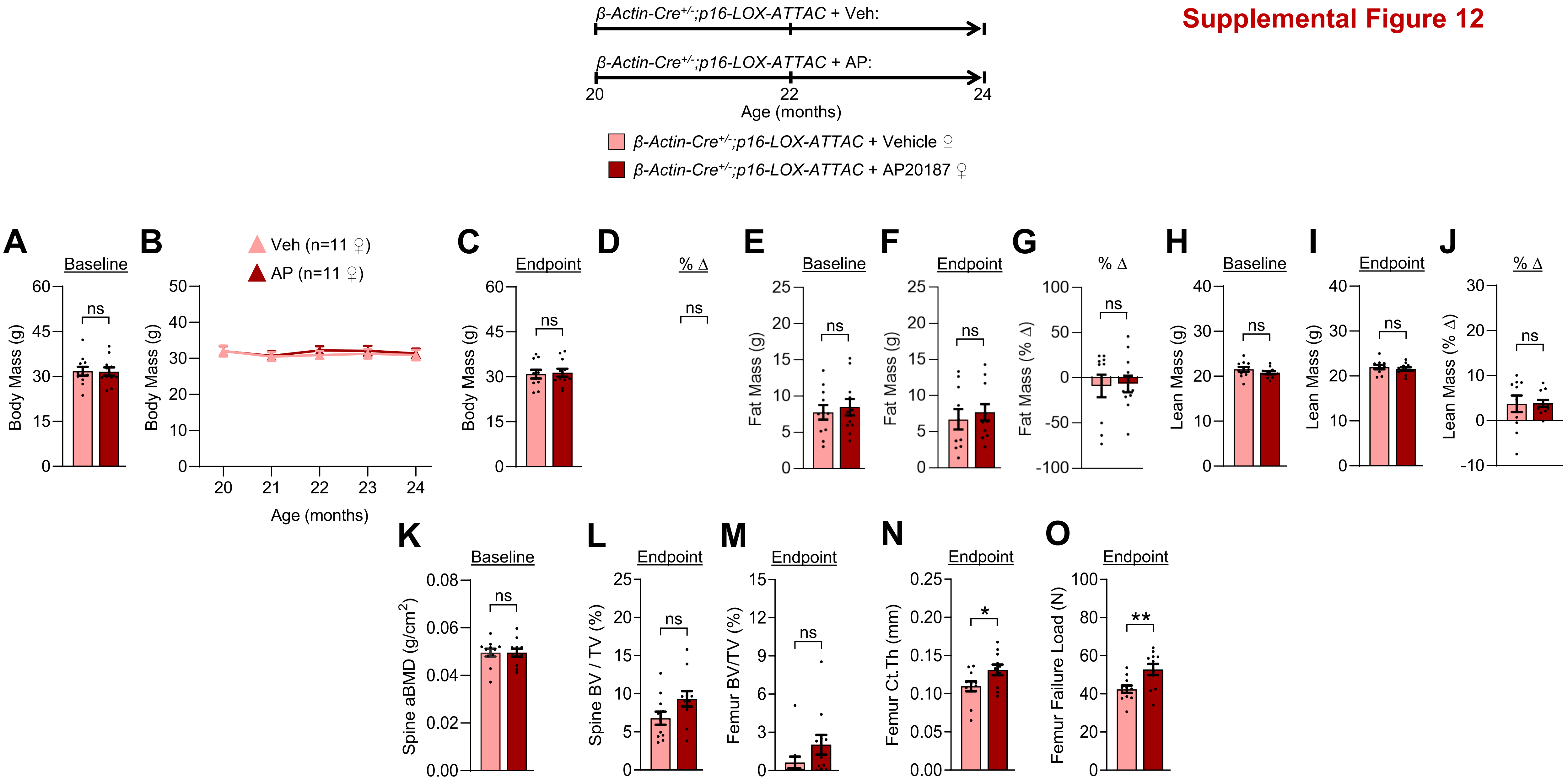

Supplemental Figure 13

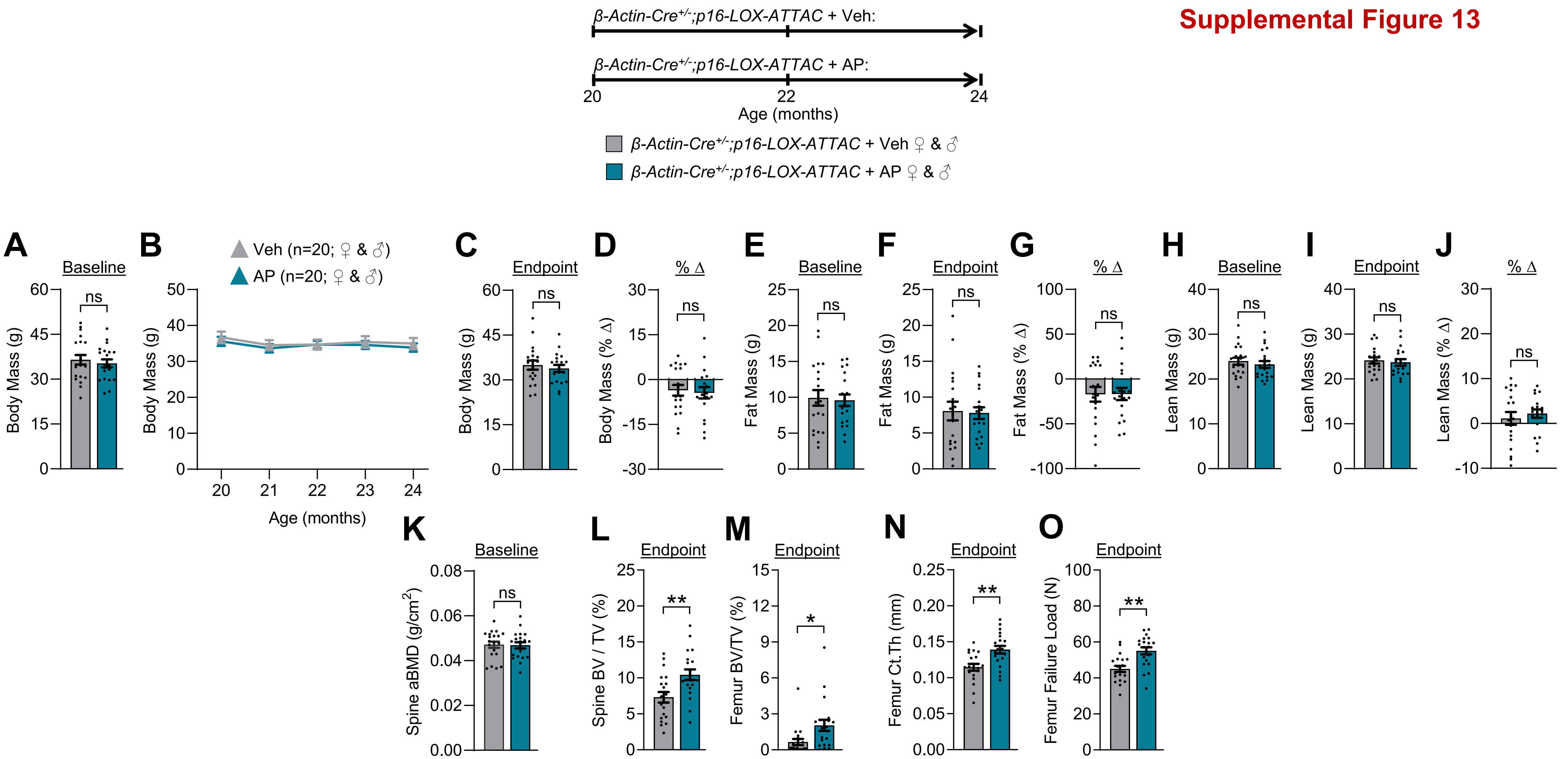

Supplemental Figure 14

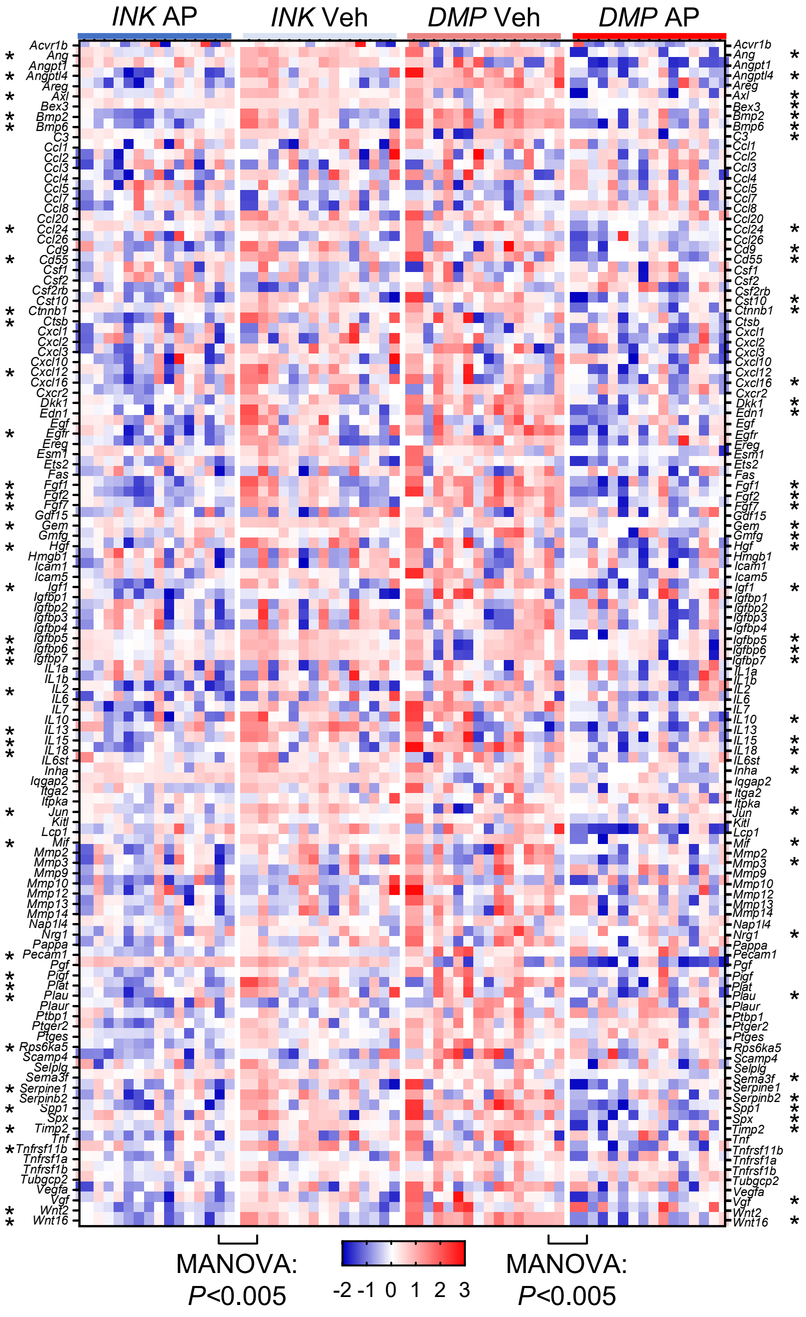

Supplemental Figure 15

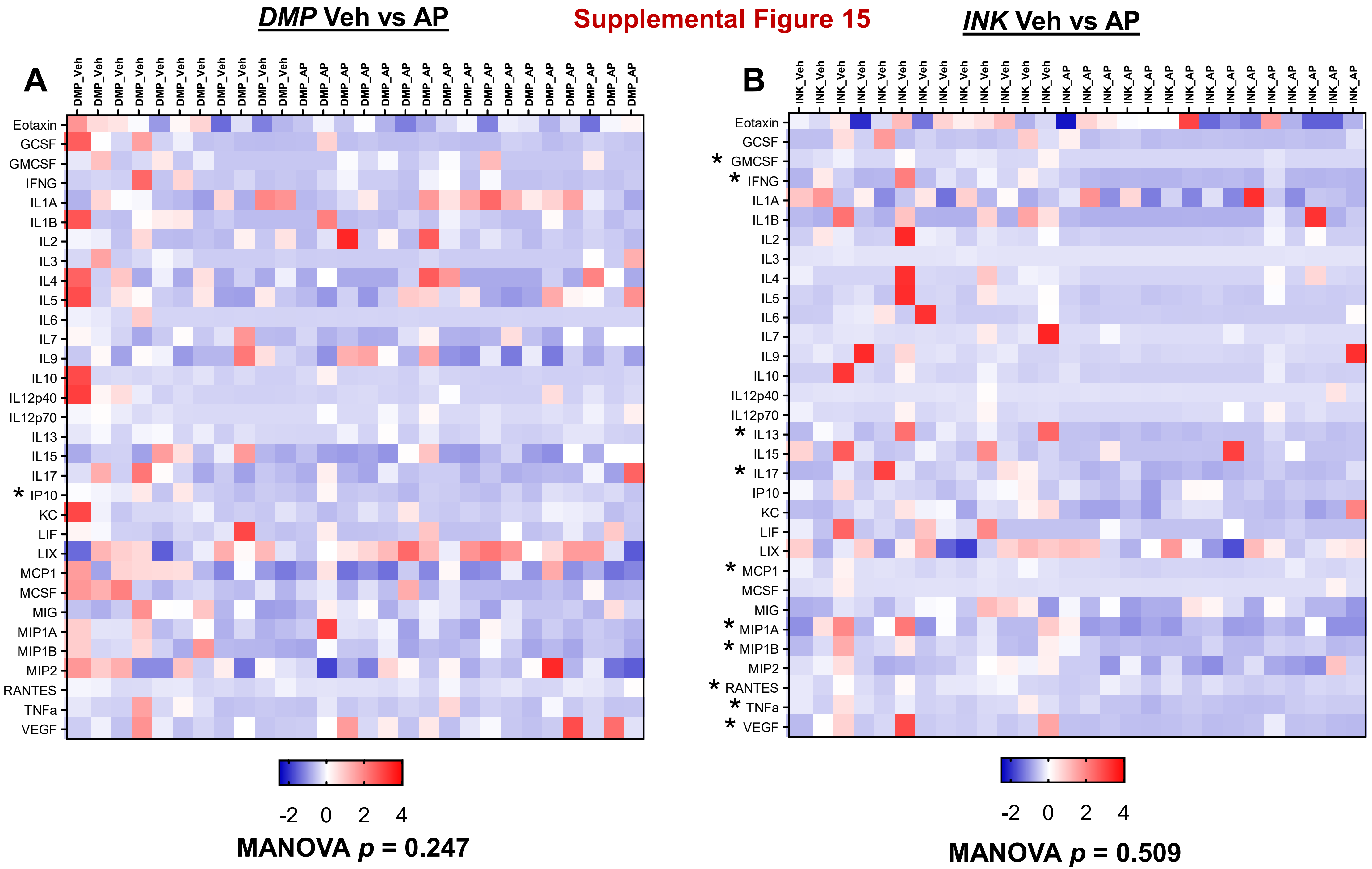

***DMP* Veh vs AP** **Supplemental Figure 16** ***INK* Veh vs AP**

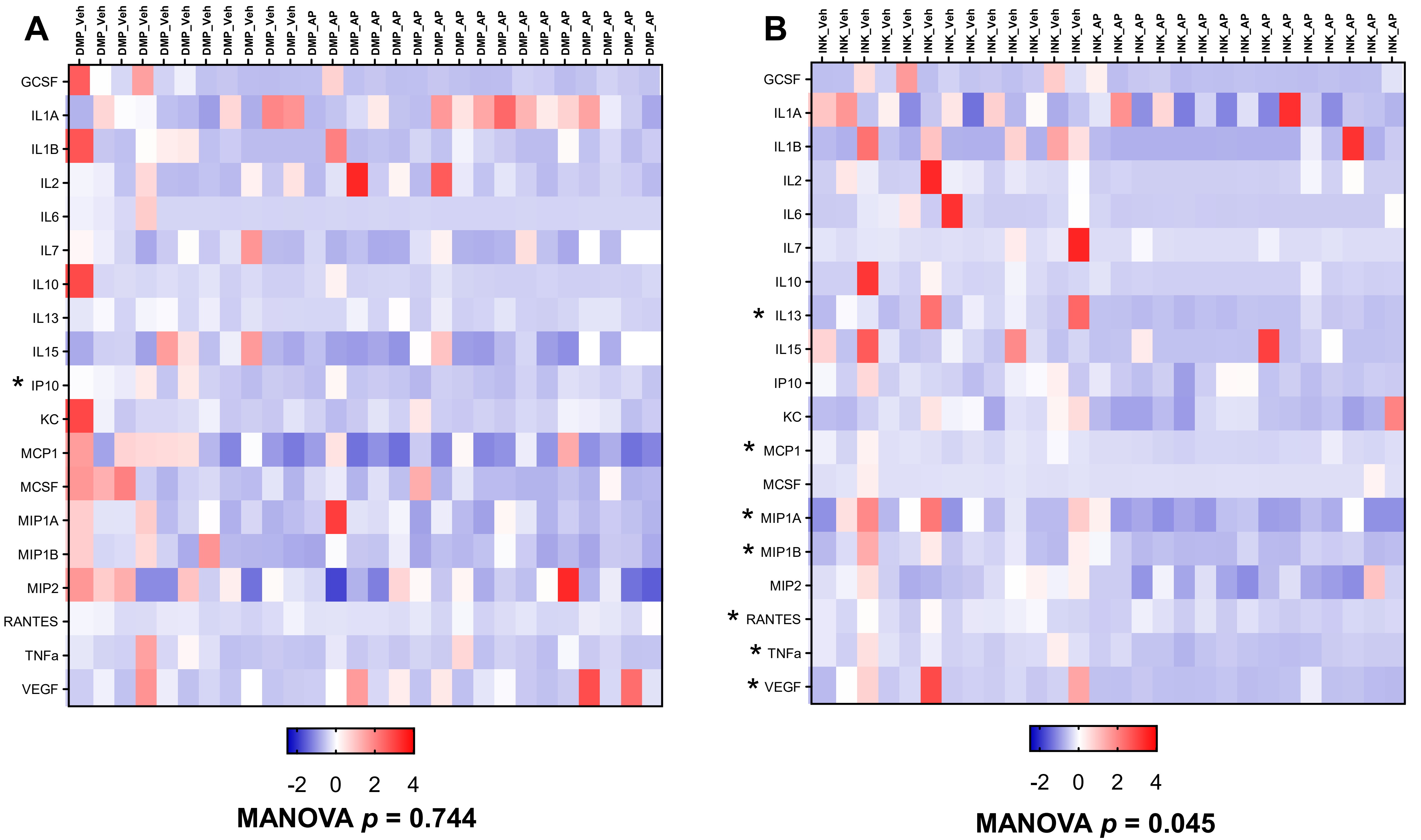

Supplemental Figure 17

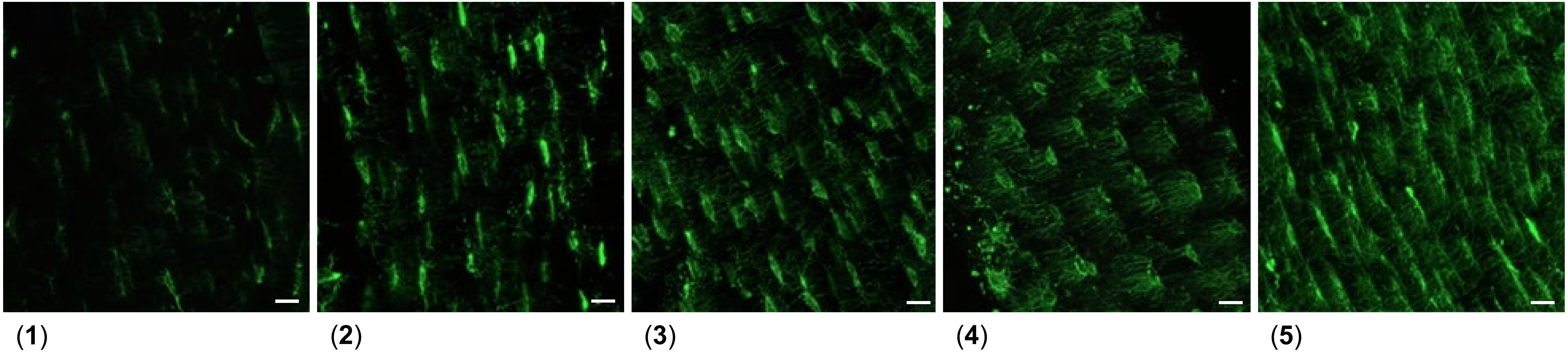

Supplemental Table 1

**Figure 1: Group × Sex Interaction *P*-values**
Fig. 1H – Bone (OCY) = 0.636
Fig. 1H – BMHCs = 0.296
Fig. 1H – Brain = 0.864
Fig. 1H – Fat = 0.894
Fig. 1H – Heart = 0.113
Fig. 1H – Kidney = 0.133
Fig. 1H – Liver = 0.508
Fig. 1H – Skel. Muscle = 0.158
Fig. 1H – Spleen = 0.706
Fig. 1I – ≥1 OCY = 0.746
Fig. 1I – ≥2 OCY = 0.115
Fig. 1I – ≥3 OCY = 0.107
Fig. 1J – ≥1 OB = 0.286
Fig. 1J – ≥2 OB = 0.091
Fig. 1J – ≥3 OB = 0.251
Fig. 1L – Fat SA-β Gal = 0.603
**Figure 3: Group × Sex Interaction *P*-values**
Fig. 3B = 0.361
Fig. 3C = 0.624
Fig. 3E = 0.252
Fig. 3F = 0.817
Fig. 3H = 0.139
Fig. 3I = 0.812
Fig. 3J = 0.333
Fig. 3K = 0.854
Fig. 3L = 0.482
Fig. 3M = 0.400
Fig. 3N = 0.821
Fig. 3O = 0.420
Fig. 3Q = 0.307
Fig. 3R = 0.755
Fig. 3S = 0.102
Fig. 3T = 0.239
**Figure 4: Group × Sex Interaction *P*-values**
Fig. 4B = 0.532
Fig. 4C = 0.789
Fig. 4E = 0.673
Fig. 4F = 0.296
Fig. 4H = 0.700
**Figure 5: Group × Sex Interaction *P*-values**

Fig. 5A *DMP* AP vs *INK* AP = 0.680
Fig. 5D *INK Sost* = 0.898
Fig. 5D *DMP Sost* = 0.788
Fig. 5E *INK Rankl* = 0.836
Fig. 5E *DMP Rankl* = 0.952
**Suppl. Fig. 4: Group × Sex Interaction *P*-values**
Suppl. Fig. 4A = 0.991
Suppl. Fig. 4B = 0.333; 0.653; 0.753
Suppl. Fig. 4C = 0.294
Suppl. Fig. 4D = 0.212
Suppl. Fig. 4E = 0.992
Suppl. Fig. 4F = 0.524
Suppl. Fig. 4G = 0.659
Suppl. Fig. 4H = 0.176
**Suppl. Fig. 9: Group × Sex Interaction *P*-values**
Suppl. Fig. 9B = 0.819
Suppl. Fig. 9C = 0.686
Suppl. Fig. 9D = 0.163
Suppl. Fig. 9E = 0.481
Suppl. Fig. 9F = 0.804
Suppl. Fig. 9G = 0.862
Suppl. Fig. 9H = 0.295
Suppl. Fig. 9I = 0.991
Suppl. Fig. 9J = 0.609
Suppl. Fig. 9K = 0.542
**Suppl. Fig. 10: Group × Sex Interaction *P*-values**
Suppl. Fig. 10B – Bone (OCY) = 0.836
Suppl. Fig. 10B – BMHCs = 0.296
Suppl. Fig. 10B – Brain = 0.189
Suppl. Fig. 10B – Fat = 0.096
Suppl. Fig. 10B – Heart = 0.396
Suppl. Fig. 10B – Kidney = 0.218
Suppl. Fig. 10B – Liver = 0.835
Suppl. Fig. 10B – Skel. Muscle = 0.278
Suppl. Fig. 10B – Spleen = 0.568
**Suppl. Fig. 13: Group × Sex Interaction *P*-values**
Suppl. Fig. 13A = 0.397
Suppl. Fig. 13B = 0.356; 0.305; 0.254
Suppl. Fig. 13C = 0.450
Suppl. Fig. 13D = 0.979
Suppl. Fig. 13E = 0.308
Suppl. Fig. 13F = 0.411

Suppl. Fig. 13G = 0.453
Suppl. Fig. 13H = 0.908
Suppl. Fig. 13I = 0.602
Suppl. Fig. 13J = 0.887
Suppl. Fig. 13K = 0.978
Suppl. Fig. 13L = 0.484
Suppl. Fig. 13M = 0.937
Suppl. Fig. 13N = 0.597
Suppl. Fig. 13O = 0.810
**Suppl. Fig. 14: Group × Sex Interaction *P*-values**
Suppl. Fig. 14 *INK* Veh vs AP = 0.428
Suppl. Fig. 14 *DMP* Veh vs AP = 0.830
**Suppl. Fig. 15: Group × Sex Interaction *P*-values**
Suppl. Fig. 15A = 0.346
Suppl. Fig. 15B = 0.999
**Suppl. Fig. 16: Group × Sex Interaction *P*-values**
Suppl. Fig. 16A = 0.793
Suppl. Fig. 16B = 0.893

Supplemental Methods

Doxorubicin (DoxR)-induced senescence in vivo study. For the DoxR *in vivo* validation experiment, 4-month-old *E2a-Cre*$^{+/-}$*;p16-LOX-ATTAC* mice were randomized to one of three groups: (i) Vehicle (Veh); (ii) DoxR (10 mg/kg) + Veh; or (iii) DoxR (10 mg/kg) + AP (10 mg/kg, twice weekly) for 24 days. DoxR (Santa Cruz) was administered on days 0 and 10. This dosing protocol has been previously shown to induce cellular senescence in young adult mice (1). Following sacrifice, mouse livers were obtained for RT-qPCR analysis of senescence markers.

Cell transplantation. Mouse ear primary fibroblasts were isolated, as described (2, 3). To induce senescence in cells to be transplanted (as detailed previously (2, 3)), cells in culture were exposed to 10 Gy cesium irradiation (IR), which consistently causes the majority (>85%) of cells to become senescent 20 days following IR (2, 3). Following trypsinization, control (CON) or senescent (SEN) cells were collected and pellets were washed with PBS. For transplantation, cells were resuspended in 150 µL of PBS and young adult (4-month-old) male C57BL/6 WT mice were (following isofluorane anesthesia) injected intraperitoneally (i.p.) using a 22-G needle, as described (3).

Mouse harvests and tissue collections. Mice were anesthetized with ketamine/xylazine and blood was collected *via* cardiac puncture at time of death (*i.e.* morning) and stored at -80°C. Subsequently, mice were euthanized and cells/tissues (bone marrow hematopoietic stem cells [BMHCs], bone [osteocyte-enriched or whole-bone], brain, fat, heart, kidney, liver, skeletal muscle, spleen) were immediately dissected for further phenotyping. Soft tissues were immediately homogenized (Tissue Tearor; Cole Parmer, Court Vernon Hills, IL) in lysis buffer (QIAzol; Qiagen, Valencia, CA) and then stored at -80°C for later extraction of RNA (see *RT-qPCR analyses*). For harvests of long bones (femurs/tibiae) and vertebrae, muscle and connective tissues were removed. The right femur and L_{4-6} lumbar vertebrae were stored in EtOH for later micro-CT phenotyping (see *Skeletal assessments*). The left femur and thoracic

vertebrae used for osteocyte-enriched cell preparations, as described in detail (4). As shown previously (4), the remaining cell preparation represents a highly enriched population of osteocytes used for RT-qPCR analyses. The proximal and distal metaphyses of the tibiae were cut and whole-bone samples prepared and then immediately homogenized (Tissue Tearor; Cole Parmer, Court Vernon Hills, IL) in QIAzol lysis reagent (Qiagen, Valencia, CA). Bone marrow cells were pooled and treated with 1 x red blood cell (RBC) lysis buffer solution (eBioscience, San Diego, CA) for 5 min at room temperature (RT). Following a brief centrifugation and resuspension in FACS buffer, the resulting BMHCs were lysed with QIAzol reagent (Qiagen, Valencia, CA) and store at -80°C.

Biochemical assays. As described previously by our group (5), the serum bone formation marker P1NP (amino-terminal propeptide of type I collagen; ng/mL) was measured using the Rat/Mouse P1NP enzyme immunoassay (EIA) kit (interassay coefficient of variation [CV] <10%), whereas the serum bone resorption marker CTx (cross-linked C-telopeptide of type I collagen; ng/mL) was measured by the RatLaps Rat/Mouse CTx EIA kit (interassay CV <10%). Kits were purchased from Immuno Diagnostic Systems (IDS, Scottsdale, AZ). All biochemical assays were performed in a blinded fashion.

Multiplex protein analyses. Luminex xMAP technology was used to quantify cytokines and chemokines, which was performed with multiplex kits from Millipore and multiplexing analysis on the Luminex 100 system (Luminex) by Eve Technologies Corp. Cytokine data imputation was performed as detailed previously (6). For all proteins, more than 80% of the samples were within the detectable range. Undetectable targets were assigned a value of half of the lowest value as described previously (6).*Body composition assessments.* Body mass (g) was recorded at baseline and monthly thereafter on all mice. At baseline and study endpoint, body composition (total body lean and fat mass) was assessed in non-anesthetized, conscious mice by quantitative Echo magnetic resonance imaging (EchoMRI-100, Houston, TX), as described (5, 7). All assessments were performed in a blinded fashion.

Skeletal assessments. All bone image acquisition and analysis were performed in a blinded fashion. *In vivo* areal bone mineral density (aBMD; g/cm^2) of the lumbar spine (L_1-L_4) was measured at baseline (see Figure Legends) by dual-energy X-ray absorptiometry (DXA) using a Lunar PIXImus densitometer (software version 1.44.005; Lunar Corp., Madison, WI); mice were randomized to treatments using a block design to ensure groups were well-matched for baseline BMD. All micro-computed tomography (µCT) imaging and analyses were performed on *ex vivo* bones (vertebrae and femur), as previously described by our laboratory (8). More specifically, quantitative measures of bone microarchitectural parameters of the right femur (distal metaphysis and mid-shaft diaphysis) as well as the lumbar vertebrae (L_5) were performed using the following scanner settings: 55 kVp, 10.5 µm voxel size, 21.5 diameter, 145mA, 300 ms integration time. From the manufacturer's protocols, trabecular bone volume fraction (BV/TV; %) was measured at the lumbar spine (200 slices) and distal metaphysis (100 slices) of the right femur. In addition, at the distal metaphysis (50 slices) and mid-diaphysis (50 slices) of the right femur, cortical thickness (Ct.Th; mm), endocortical circumference (EC; mm), and periosteal circumference (PC; mm) were assessed. In addition, bone strength at the proximal metaphysis (50 slices) and mid-diaphysis (50 slices) of the right tibia was simulated *via* micro-finite element analysis (µFEA) to assess failure load (N) using the manufacturer's software (Scanco Medical AG, Basserdorf, Switzerland; Finite Element-Software Version 1.13), as described (8).

Bone histomorphometry assessments. All histomorphometry analyses were performed in blinded fashion as previously described by our group (5, 8). The non-decalcified right femur was embedded in methyl-methacrylate (MMA) and sectioned. For static analyses, sections were stained for tartrate-resistant acid phosphatase (TRAP) activity to evaluate osteoclast numbers per bone perimeter (N.Oc/B.Pm, /mm) or with Masson's Trichrome stain to assess osteoblast numbers per bone perimeter (N.Ob/B.Pm, /mm) as well as total osteocyte numbers per bone area and percentage (%) of OCY empty lacunae. For dynamic fluorochrome analyses, mice were injected i.p. with Alizarin Red (0.1 mL/mouse, 7.5 mg/mL, Sigma-Aldrich, Saint Louis, MO)

and calcein (0.1 mL/mouse, 2.5 mg/mL, Sigma-Aldrich, Saint Louis, MO) on days nine and two, respectively, prior to euthanasia. Unstained sections were used to evaluate bone formation rate per bone surface (BFR/BS, $mcm^3/mcm^2/d$). Bone marrow adipocyte parameters were determined as previously described by our group (8). Briefly, adipocyte numbers were measured by tracing out individual adipocytes in all the fields analyzed. Histomorphometry measurements and calculations were conducted using the Osteomeasure Analysis system (Osteometrics, Atlanta, GA).

Conditioned medium generation. Mouse ear clippings were collected from C57BL/6 mice for isolation of primary fibroblasts, as described (2). Cellular senescence was induced in primary fibroblasts by 10 Gy of cesium irradiation, as described (2) and conditioned medium (CM) was generated, as described (3). For collection, murine non-proliferating control (CON) or senescent (SEN) fibroblasts were washed three times with PBS and cultured in CM for 24 hrs. Parallel control cultures were not irradiated (IR).

In vitro osteoblast differentiation. BMSCs were plated at a density of 1×10^4 cells per cm^2 in α-MEM without ascorbic acid. After reaching confluency (~48 hr), media was replaced, and cells were cultured for an additional 72 hrs. Differentiation media consisted of α-MEM containing ascorbic acid with the addition of 50 µg/mL ascorbic acid and 10-mM β-glycerophosphate. Cells were cultured in the presence of 20% CON or SEN CM, as described (8). Differentiation media and CM treatments were replaced every 3 or 4 days until day 7 or 14. Cells were fixed with 1% PFA and then stained with Alizarin Red S (Sigma-Aldrich) to assess osteoblast mineralization. Cultures were stained for 15 min, washed with H_2O, and dried before obtaining images. Alizarin Red stain was eluted with 10% cetylpyridinium chloride in NaP buffer and absorbance was assessed at 540 nm.

In vitro adipocyte differentiation. Bone marrow stromal cells (BMSCs) were plated at a density of 1×10^4 cells per cm^2 with media (αMEM/10% FBS) supplemented with 0.5 µg/mL insulin, 0.5 mM IBMX, 200 µm indomethacin, and 1 µm dexamethasone. Cells were cultured to 80%

confluence in the presence of 20% CON or SEN CM, as described (8). Differentiation media and CM treatments were replaced every 3 or 4 days until day 14. Cells were fixed with 1% PFA, rinsed, and then stained for 30 min with 0.15% Oil Red O (Sigma) in a 55:45 mix of isopropanol and H_2O. Plates were dried before obtaining images.

TUNEL assay to detect apoptotic osteocytes. Mouse tibiae were collected and fixed in 4% phosphate-buffered formalin for 72 hr, decalcified in 10% EDTA for two weeks. Prior to embedding and sectioning, bones were dehydrated in xylene and resuspended in paraffin wax. Sectioned slides were deparaffinized through a series of xylene, EtOH, and 1x phosphate buffered saline (PBS) washes, followed by a 15 min incubation in 20µg/mL of proteinase K. The ApopTag® Red in Situ Apoptosis Detection Kit (EMD Millipore, Billerica, MA) was then used according to the manufacturer's instructions to detect apoptotic osteocytes. Next, slides were equilibrated in buffer, followed by a 1 hr incubation at 37°C with TdT enzyme in buffer, which was then blocked by washing slides with stop/wash solution, followed by rinsing with 1x PBS. Cells were conjugated to rhodamine using anti-digoxigenin in blocking solution with a 30 min incubation in a humidified chamber at RT. Following incubation, sections were mounted and coverslipped with ProLong™ Gold antifade reagent with DAPI prior to imaging.

Osteocyte lacunocanicular network (LCN) analysis. Femurs were fixed in 4% phosphate-buffered formalin for 72 hrs and decalcified in 10% EDTA for two weeks prior to frozen embedding. Bones were embedded in an orientation that permitted acquisition of transverse sections representative of full osteocyte network depth, with 50 µm cryosections spanning both the distal metaphysis and diaphysis. Samples were then incubated overnight at 4°C in 165nM AlexaFluorTM 488 phalloidin, followed by three 1x PBS washes. ProLong™ Gold antifade reagent with DAPI was then used to mount and coverslip each section prior to imaging. An LSM 780 inverted confocal microscope was used to obtain Z-stacks of the osteocytes and adjoining canicular networks with a H_2O-immersed 63x objective. Three images of OCY canicular networks at both the distal metaphyseal and diaphyseal regions were obtained and analyzed in

a blinded fashion. Prior to analysis, Z-stacks were created with ImageJ software. Images were scored using a semiquantitative 1-5 scale, where one represented a poorly connected OCY LCN whereas five represented a high quality OCY LCN, as shown in Supplemental Figure 17.

Immunostaining for FLAG. Bone marrow stromal cells (BMSCs) were obtained from 4-month-old *p16-LOX-ATTAC mice* (non-crossed controls) or *CMV-Cre+/-;p16-LOX-ATTAC* (recombined). Cells were plated and then subjected to either irradiation (IR, 10 Gy) or Sham-IR (non-IR controls) and then cultured for 5 days. Separate plates were used for RT-qPCR analysis of *EGFP* mRNA expression (see below) versus immunostaining. As described (9), transgene expression was verified using immunostaining for FLAG (Sigma, Cat. # F1804; RRID: AB_262044) following permeabilization with Triton X 0.3% solution, washes with PBS + 1% NDS, and overnight primary antibody incubation. This was followed by incubations for 1 hr with secondary antibodies coupled to Cy5 and DAPI for nuclear staining.

Telomere-Associated Foci (TAF). To measure osteocyte or osteoblast cellular senescence, as described previously (5), the TAF assay was performed on non-decalcified methacrylate-embedded sections of tibia isolated from young adult (6-month-old) and old (24-month-old) *C57BL/6 WT* mice, old (24-month-old) *DMP1-Cre$^{+/-}$;p16-LOX-ATTAC* mice treated with either vehicle or AP20187, or young male *C57BL/6 WT* mice transplanted with either Control or Senescent (SEN) fibroblasts. As adapted from a previous protocol (10), bone sections were de-plasticized and hydrated with gradients of EtOH followed by washes in H_2O and PBS. Antigen retrieval was achieved by Tris-EDTA incubation (pH 9.0) at 95°C for 15 min. Following cooldown and then hydration with H_2O and PBS (0.5% Tween-20/0.1% Triton X-100), slides were placed in blocking buffer (1:60 normal goat serum; Vector Laboratories; S-1000; in 0.1% BSA/PBS) at RT for 30 min. After dilution in blocking buffer, primary antibody γ-H2AX (1:200; anti–γ-H2A.X rabbit monoclonal antibody, Cell Signaling Technology; 9718) was incubated at 4°C overnight. The next morning, slides were washed with PBS (0.5% Tween-20/0.1% Triton X-100), PBS alone, and then incubated with secondary goat, biotinylated anti-rabbit antibody (1:200; Vector

Laboratories; BA-1000) in blocking buffer at 4°C for 30 min. Slides were next washed with PBS (0.5% Tween-20/0.1% Triton X-100), PBS alone, and then incubated with tertiary antibody (1:500; Cy5 Streptavidin, Vector Laboratories; SA-1500) in PBS at 4°C for 60 min. Subsequently, slides were washed (three times) with PBS, followed by fluorescence *in situ* hybridization (FISH) for detection of TAF. Briefly, following 4% paraformaldehyde (PFA) cross-linking for 20 min, sections were washed with PBS (three times, five min each) and dehydrated in graded (70%, 90%, 100%) ice-cold EtOH (three min each). Sections were then dried/denatured for 10 min at 80°C with hybridization buffer that contained 0.1 M Tris (pH 7.2), 25 mM $MgCl_2$, 70% formamide (Millipore Sigma), 5% blocking reagent (Roche), and 1.0 μg/mL of Cy3-labeled telomere-specific (CCCTAA) peptide nucleic acid probe (TelC-Cy3, Panagene Inc.; F1002). This was followed by hybridization in a humidified dark room at RT for two hrs. Sections were then washed and mounted with VECTASHIELD DAPI-containing mounting medium (Life Technologies) prior to acquisition of images and their analysis, which was performed in a blinded fashion. TAF number per osteocyte or osteoblast was quantified by examining overlap (yellow) of signals from the telomere probe (red) with the γ-H2AX (green) – *i.e.* phosphorylated C-terminal end of histone H2A.X, thus marking double-strand DNA breaks. The average number of TAF per osteocyte (OCY) or osteoblast (OB) was quantified using FIJI (ImageJ software; NIH, https://imagej.nih.gov/ij/), whereas the percentage (%) of TAF+ OCYs or OBs for each mouse was calculated using the following criteria: % of cells with ≥1 TAF, % of cells with ≥2 TAF, and % of cells with ≥3 TAF.

Real-Time quantitative polymerase chain reaction (RT-qPCR) analyses. Gene expression measurements were performed using RT-qPCR, as described (5, 8). Briefly, cells or tissues (extracted as described in *Mouse harvest and collections*) were immediately homogenized in QIAzol Lysis Reagent (QIAGEN, Valencia, CA) and stored at -80°C for subsequent RNA extraction, standard cDNA synthesis, and targeted gene expression assessments of mRNA levels by RT-qPCR. Total RNA was isolated from QIAzol-stored samples using RNeasy Mini

Columns (QIAGEN, Valencia, CA). DNase treatment was applied to degrade contaminating genomic DNA using an on-column RNase-free DNase solution (QIAGEN, Valencia, CA). RNA quantity and purity were confirmed with a Nanodrop spectrophotometer (Thermo Scientific, Wilmington, DE). Reverse transcriptase was performed using the High-Capacity cDNA Reverse Transcription Kit (Applied Biosystems by Life Technologies, Foster City, CA). PCR reactions were run using the ABI Prism 7900HT Real Time System (Applied Biosystems, Carlsbad, CA) with SYBR green (QIAGEN, Valencia, CA) as the detection method. Murine primer sequences were designed using Primer Express Software Version 3.0 (Applied Biosystems). As done previously (5, 8), input RNA variations were normalized using a panel of five reference genes (*Actb, Hprt, Polr2a, Tbp, Tub1a*) from which the three most stable housekeeping genes were determined by the geNorm algorithm (http://medgen.ugent.be/~jvdesomp/genorm/) (11, 12), followed by the PCR Miner algorithm (13) to adjust for variability in amplification efficiency. For each sample, the median cycle threshold (Ct) of each gene (run in triplicate) was normalized to the geometric mean of the median Ct of the three most stable reference genes, which was determined using the following geNorm algorithm: 2(reference Ct – gene of interest Ct). The ΔCt for each gene was used to calculate the relative mRNA expression changes for each sample. Genes with Ct values >35 were considered to be Not Expressed (NE), as done previously (4).

Supplemental References

1. Baar MP, Brandt RMC, Putavet DA, Klein JDD, Derks KWJ, Bourgeois BRM, et al. Targeted Apoptosis of Senescent Cells Restores Tissue Homeostasis in Response to Chemotoxicity and Aging. *Cell.* 2017;169(1):132-47 e16.
2. Ogrodnik M, Miwa S, Tchkonia T, Tiniakos D, Wilson CL, Lahat A, et al. Cellular senescence drives age-dependent hepatic steatosis. *Nat Commun.* 2017;8:15691.
3. Xu M, Bradley EW, Weivoda MM, Hwang SM, Pirtskhalava T, Decklever T, et al. Transplanted Senescent Cells Induce an Osteoarthritis-Like Condition in Mice. *J Gerontol A Biol Sci Med Sci.* 2016.
4. Farr JN, Fraser DG, Wang H, Jaehn K, Ogrodnik MB, Weivoda MM, et al. Identification of Senescent Cells in the Bone Microenvironment. *J Bone Miner Res.* 2016;31(11):1920-9.
5. Eckhardt BA, Rowsey JL, Thicke BS, Fraser DG, O'Grady KL, Bondar OP, et al. Accelerated osteocyte senescence and skeletal fragility in mice with type 2 diabetes. *JCI Insight.* 2020;5(9).
6. Schafer MJ, Zhang X, Kumar A, Atkinson EJ, Zhu Y, Jachim S, et al. The senescence-associated secretome as an indicator of age and medical risk. *JCI Insight.* 2020;5(12).
7. Farr JN, Rowsey JL, Eckhardt BA, Thicke BS, Fraser DG, Tchkonia T, et al. Independent Roles of Estrogen Deficiency and Cellular Senescence in the Pathogenesis of Osteoporosis: Evidence in Young Adult Mice and Older Humans. *J Bone Miner Res.* 2019;34(8):1407-18.
8. Farr JN, Xu M, Weivoda MM, Monroe DG, Fraser DG, Onken JL, et al. Targeting cellular senescence prevents age-related bone loss in mice. *Nat Med.* 2017;23(9):1072-9.
9. Aguayo-Mazzucato C, Andle J, Lee TB, Jr., Midha A, Talemal L, Chipashvili V, et al. Acceleration of beta Cell Aging Determines Diabetes and Senolysis Improves Disease Outcomes. *Cell Metab.* 2019;30(1):129-42 e4.
10. Hewitt G, Jurk D, Marques FD, Correia-Melo C, Hardy T, Gackowska A, et al. Telomeres are favoured targets of a persistent DNA damage response in ageing and stress-induced senescence. *Nat Commun.* 2012;3:708.
11. Radonic A, Thulke S, Mackay IM, Landt O, Siegert W, and Nitsche A. Guideline to reference gene selection for quantitative real-time PCR. *Biochem Biophys Res Commun.* 2004;313:856-62.
12. Vandesompele J, De Preter K, Pattyn F, Poppe B, Van Roy N, De Paepe A, et al. Accurate normalization of real-time quantitative RT-PCR data by geometeric averaging of multiple internal control genes. *Genome Biol.* 2002;3:research0034.1-0-34.11.
13. Zhao S, and Fernald RD. Comprehensive algorithm for quantitative real-time polymerase chain reaction. *J Comput Biol.* 2005;12(8):1047-64.
